# Supplementary material for: Hypothesis-generating analysis of the impact of non-damaging metabolic acidosis on the transcriptome of different cell types: Integrated stress response (ISR) modulation as general transcriptomic reaction to non-respiratory acidic stress?
Source: PLoS One. 2023 Aug 25;18(8):e0290373. doi: 10.1371/journal.pone.0290373 (PMC10456223; doi:10.1371/journal.pone.0290373)
Supplement: S1 File — (PDF) [file pone.0290373.s001.pdf]

INGENUITY
PATHWAY ANALYSIS

Analysis Name: SA1A - Monoculture - HK2 (with homologs) - 2023-02-16 10:59 vorm.
Analysis Creation Date: 2023-02-16
Build version: exported
Content version: 84978992 (Release Date: 2022-11-27)

Experiment Metadata

Name	Value

Analysis Settings

Reference set: Ingenuity Knowledge Base (Genes Only)
Relationship to include: Direct and Indirect
Includes Endogenous Chemicals
Optional Analyses: My Pathways My List

Filter Summary:
Consider only molecules and/or relationships where
(species = Uncategorized OR Human OR Mouse OR Rat) AND
(confidence = Experimentally Observed) AND
(tissues/cell lines = Other Dendritic cells OR Corpus Callosum OR Pancreas OR Other Melanoma Cell Lines OR Other Cell Line OR Hematopoietic progenitor cells OR Murine NKT cells OR SN12C OR U251 OR Central memory helper T cells OR Stromal cells OR White Matter OR Neutrophils OR Placenta OR Other Lymphoma Cell Lines OR Cornea OR NCI-H23 OR Other Smooth muscle cells OR Cos-7 cells OR SF-

(c) 2000-2023 QIAGEN. All rights reserved.

268 OR CD34+ cells OR Th2 cells OR Vd2 Gamma-delta T cells OR Memory T lymphocytes not otherwise specified OR Chondrocytes OR Skeletal Muscle OR Striatum OR HT29 OR Central memory cytotoxic T cells OR Trigeminal Ganglion OR J-774A.1 OR A549-ATCC OR Nucleus Accumbens OR Plasma cells OR A375 OR Granule cells OR SK-MEL-2 OR HepG2 OR Forestomach OR Amygdala OR Cardiomyocytes OR Granulocytes not otherwise specified OR SF-295 OR CD56dim NK cells OR Spleen OR RBL-2H3 OR CAKI-1 OR MCF7 OR Activated helper T cells OR Granulosa cells OR Activated Vd1 Gamma-delta T cells OR T lymphocytes not otherwise specified OR Colon Cancer Cell Lines not otherwise specified OR Other Breast Cancer Cell Lines OR Brainstem OR Cells not otherwise specified OR Peripheral blood monocytes OR Other Monocyte-derived dendritic cells OR NB4 OR Medulla Oblongata OR WEHI-231 OR Cytotoxic T cells OR BT-549 OR Lymphoma Cell Lines not otherwise specified OR Beta islet cells OR NCI-H332M OR Other Colon Cancer Cell Lines OR Other Neuroblastoma Cell Lines OR Other Stem cells OR Caudate Nucleus OR Melanocytes OR Other Cells OR Other Ovarian Cancer Cell Lines OR Monocyte-derived dendritic cells not otherwise specified OR Other Organ Systems OR Peripheral blood lymphocytes OR Other Hepatoma Cell Lines OR 3T3-L1 cells OR Other Bone marrow cells OR Fibroblasts OR HOP-92 OR Salivary Gland OR Large Intestine OR CNS Cell Lines not otherwise specified OR Bone marrow-derived macrophages OR Effector memory cytotoxic T cells OR Naive helper T cells OR UACC-62 OR OVCAR-8 OR OVCAR-4 OR MEF cells OR SK-MEL-5 OR Microvascular endothelial cells OR Other Prostate Cancer Cell Lines OR 786-0 OR Granule Cell Layer OR Lymphocytes not otherwise specified OR NCI-ADR-RES OR Dendritic cells not otherwise specified OR Stomach OR CD56bright NK cells OR Other Kidney cell lines OR Min6 OR Purkinje cells OR Activated CD56bright NK cells OR Brain OR Langerhans cells OR Mesenchymal stem cells OR Thalamus OR J774 OR Other CNS Cell Lines OR MDA-MB-468 OR UACC-257 OR Bone marrow cells not otherwise specified OR Other Nervous System OR MDA-MB-435 OR Jurkat OR RKO OR Macrophage Cancer Cell Lines not otherwise specified OR Cell Line not otherwise specified OR U266 OR Other Memory T lymphocytes OR Skin OR Monocyte-derived macrophage OR ACHN OR Other Granulocytes OR Melanoma Cell Lines not otherwise specified OR HCT-15 OR Pheochromocytoma cell lines not otherwise specified OR MOLT-4 OR RAW 264.7 OR Lung Cancer Cell Lines not otherwise specified OR SK-N-SH OR Other Pancreatic Cancer Cell Lines OR COLO205 OR Epithelial cells not otherwise specified OR Pro-B lymphocytes OR SK-OV-3 OR Testis OR Other Neurons OR RXF-393 OR Gray Matter OR Other NK cells OR BT-474 OR Olfactory Bulb OR Prostate Gland OR P19 OR Vd1 Gamma-delta T cells OR A2780 OR HS 578T OR Cortical neurons OR Other Osteosarcoma Cell Lines OR Megakaryocytes OR Memory B cells OR Kidney OR Trachea OR U937 OR Breast Cancer Cell Lines not otherwise specified OR LOX IMVI OR RPMI-8266 OR Natural T-regulatory cells OR MG-63 OR Th17 cells OR Oocytes OR Esophagus OR Other Endothelial cells OR TK-10 OR Monocytes not otherwise specified OR 293 cells OR SNB-75 OR Caco2 cells OR EKVX OR Other Lymphocytes OR Peritoneal macrophages OR Calvaria OR Other Immune cell lines OR Other Macrophage Cancer Cell Lines OR Hep3B OR Cervical cancer cell line not otherwise specified OR Prostate Cancer Cell Lines not otherwise specified OR T47-D OR Mammary Gland OR CD4+ T-lymphocytes OR Cerebellum OR Leukemia Cell Lines not otherwise specified OR CCRF-CEM OR KM-12 OR BDCA-1+ dendritic cells OR Hepatoma Cell Lines not otherwise specified OR Other Peripheral blood leukocytes OR U87MG OR Sciatic Nerve OR Other Macrophages

(c) 2000-2023 QIAGEN. All rights reserved.

OR Immune cell lines not otherwise specified OR LNCaP cells OR MDA-MB-231 OR HeLa OR HMC-1 OR UO-31 OR Crypt OR Activated Vd2 Gamma-delta T cells OR Naive B cells OR Parietal Lobe OR Dermis OR Thyroid Gland OR Other Pheochromocytoma cell lines OR NIH/3T3 cells OR Other T lymphocytes OR Cartilage Tissue OR Other Kidney Cancer Cell Lines OR PBMCs OR Adrenal Gland OR Effector memory RA+ cytotoxic T cells OR Heart OR Smooth Muscle OR Liver OR Osteoblasts OR Bladder OR Epidermis OR Mast cells OR Small Intestine OR Osteosarcoma Cell Lines not otherwise specified OR A498 OR Other Monocytes OR Peripheral blood leukocytes not otherwise specified OR Pituitary Gland OR Blood platelets OR THP-1 OR Macrophages not otherwise specified OR Myeloid dendritic cells OR Dorsal Root Ganglion OR HCC-2998 OR Adipose OR Cerebral Ventricles OR Pyramidal neurons OR NCI-H226 OR U2OS OR Myeloma Cell Lines not otherwise specified OR Substantia Nigra OR Cerebral Cortex OR Other Myeloma Cell Lines OR M14 OR Thymus OR DU-145 OR HL-60 OR Astrocytes OR B lymphocytes not otherwise specified OR Uterus OR PC-12 cells OR Vascular smooth muscle cells OR Th1 cells OR HuH7 OR Microglia OR Choroid Plexus OR Other Immune cells OR Spinal Cord OR Mature monocyte-derived dendritic cells OR Fibroblast cell lines not otherwise specified OR HCT-116 OR NT2/D1 OR Intraepithelial T lymphocytes OR Lung OR NCI-H522 OR PANC-1 OR Tissues and Primary Cells not otherwise specified OR Other Mononuclear leukocytes OR SW-480 OR BDCA-3+ dendritic cells OR Smooth muscle cells not otherwise specified OR SK-MEL-28 OR BA/F3 OR Ovarian Cancer Cell Lines not otherwise specified OR Other Epithelial cells OR Putamen OR Neurons not otherwise specified OR Effector T cells OR Other Tissues and Primary Cells OR Neuroblastoma Cell Lines not otherwise specified OR Pancreatic Cancer Cell Lines not otherwise specified OR MDA-MB-361 OR SR OR Bone marrow-derived dendritic cells OR Lens OR MALME-3M OR HOP-62 OR HEL OR Activated CD56dim NK cells OR Nervous System not otherwise specified OR Plasmacytoid dendritic cells OR Swiss 3T3 cells OR Endothelial cells not otherwise specified OR Hepatocytes OR Ventricular Zone OR PC-3 OR Immature monocyte-derived dendritic cells OR Immune cells not otherwise specified OR Retina OR Mononuclear leukocytes not otherwise specified OR Organ Systems not otherwise specified OR Other Lung Cancer Cell Lines OR Subventricular Zone OR H460 OR K-562 OR INS-1 OR Kidney cell lines not otherwise specified OR Hippocampus OR Teratocarcinoma Cell Lines not otherwise specified OR Sertoli cells OR Kidney Cancer Cell Lines not otherwise specified OR Effector memory helper T cells OR SF-539 OR Adipocytes OR MDA-N OR Eosinophils OR NK cells not otherwise specified OR Splenocytes OR OVCAR-3 OR SW-620 OR Ovary OR Embryonic stem cells OR HUVEC cells OR OVCAR-5 OR Hypothalamus OR Stem cells not otherwise specified OR Other Leukemia Cell Lines OR Other Teratocarcinoma Cell Lines OR Thymocytes OR Other Cervical cancer cell line OR Keratinocytes OR Pre-B lymphocytes OR Other B lymphocytes OR Other Fibroblast cell lines OR Lymph node OR IGROV1) AND
(mol. types = biologic drug OR canonical pathway OR chemical - endogenous mammalian OR chemical - endogenous non-mammalian OR chemical - kinase inhibitor OR chemical - other OR chemical - protease inhibitor OR chemical drug OR chemical reagent OR chemical toxicant OR complex OR cytokine OR disease OR enzyme OR function OR fusion gene/product OR G-protein coupled receptor OR group OR growth factor OR ion channel OR kinase OR ligand-dependent nuclear receptor OR mature microRNA OR microRNA OR other OR peptidase OR phosphatase OR transcription regulator OR translation regulator OR transmembrane receptor OR transporter) AND

(c) 2000-2023 QIAGEN. All rights reserved.

INGENUITY PATHWAY ANALYSIS

(data sources = An Open Access Database of Genome-wide Association Results OR BIND OR BioGRID OR Catalogue Of Somatic Mutations In Cancer (COSMIC) OR Chemical Carcinogenesis Research Information System (CCRIS) OR Clinical Genome Resource (ClinGen) OR ClinicalTrials.gov OR ClinVar OR Cognia OR DIP OR DrugBank OR Gene Ontology (GO) OR GVK Biosciences OR Hazardous Substances Data Bank (HSDB) OR HumanCyc OR Ingenuity Expert Findings OR Ingenuity ExpertAssist Findings OR IntAct OR Interactome studies OR MIPS OR miRBase OR miRecords OR Mouse Genome Database (MGD) OR Obesity Gene Map Database OR Online Mendelian Inheritance in Man (OMIM) OR TarBase OR TargetScan Human OR TargetScan Mouse)

Top Canonical Pathways

Name	p-value	Overlap
**Kinetochore Metaphase Signaling Pathway**	6,25E-18	21,6 % 24/111
**Cell Cycle: G2/M DNA Damage Checkpoint Regulation**	5,07E-09	22,0 % 11/50
**Mitotic Roles of Polo-Like Kinase**	1,22E-08	17,9 % 12/67
**Cell Cycle Control of Chromosomal Replication**	2,09E-07	17,9 % 10/56
**Superpathway of Cholesterol Biosynthesis**	1,70E-06	24,1 % 7/29

Top Upstream Regulators

Upstream Regulators

Name	p-value	Predicted Activation
**Eldr**	7,71E-40	Inhibited
**l-asparaginase**	1,75E-34	Activated

(c) 2000-2023 QIAGEN. All rights reserved.

INGENUITY PATHWAY ANALYSIS

**CKAP2L**	3,66E-27	Inhibited
**E2F4**	6,92E-25	Activated
**TP53**	2,80E-22	Activated

Causal Network

Name	p-value	Predicted Activation
**Eldr**	7,71E-40	Inhibited
**l-asparaginase**	1,22E-34	Activated
**CKAP2L**	3,66E-27	Inhibited
**PI 3-kinase inhibitor**	4,69E-26	Inhibited
**MSX2**	3,74E-25	Activated

Top Diseases and Bio Functions

Diseases and Disorders

Name	p-value range	# Molecules
**Cancer**	9,42E-05 - 1,09E-34	483
**Organismal Injury and Abnormalities**	9,42E-05 - 1,09E-34	484
**Endocrine System Disorders**	6,94E-05 - 7,92E-30	433
**Hematological Disease**	9,42E-05 - 2,09E-28	210
**Immunological Disease**	9,42E-05 - 2,09E-28	206

(c) 2000-2023 QIAGEN. All rights reserved.

INGENUITY PATHWAY ANALYSIS

Molecular and Cellular Functions

Name	p-value range	# Molecules
**Cell Cycle**	9,24E-05 - 1,12E-23	144
**Cellular Assembly and Organization**	8,02E-05 - 1,12E-23	144
**DNA Replication, Recombination, and Repair**	8,67E-05 - 1,12E-23	105
**Cell Death and Survival**	5,83E-05 - 7,59E-15	218
**Cellular Function and Maintenance**	7,64E-05 - 4,11E-12	97

Physiological System Development and Function

Name	p-value range	# Molecules
**Organismal Survival**	1,27E-05 - 7,25E-11	161
**Hair and Skin Development and Function**	1,13E-06 - 1,13E-06	9
**Connective Tissue Development and Function**	4,50E-05 - 3,39E-06	44
**Tissue Morphology**	9,42E-05 - 3,39E-06	50
**Organismal Development**	5,60E-05 - 6,44E-06	58

Top Tox Functions

Assays: Clinical Chemistry and Hematology

Name	p-value range	# Molecules
**Increased Levels of Blood Urea Nitrogen**	2,84E-02 - 2,84E-02	3

(c) 2000-2023 QIAGEN. All rights reserved.

INGENUITY PATHWAY ANALYSIS

**Increased Levels of Alkaline Phosphatase**	8,25E-02 - 8,25E-02	4
**Increased Levels of Red Blood Cells**	9,75E-02 - 9,75E-02	5
**Increased Levels of AST**	1,19E-01 - 1,19E-01	1
**Increased Levels of ALT**	1,37E-01 - 1,37E-01	1

Cardiotoxicity

Name	p-value range	# Molecules
**Cardiac Dysfunction**	4,35E-01 - 7,81E-03	13
**Cardiac Arteriopathy**	3,29E-01 - 8,46E-03	14
**Cardiac Enlargement**	1,00E00 - 1,46E-02	26
**Cardiac Arrythmia**	1,00E00 - 2,08E-02	13
**Cardiac Dilation**	1,00E00 - 2,08E-02	10

Hepatotoxicity

Name	p-value range	# Molecules
**Liver Hyperplasia/Hyperproliferation**	1,00E00 - 1,04E-18	266
**Hepatocellular carcinoma**	1,00E00 - 3,25E-13	104
**Liver Steatosis**	2,21E-01 - 9,59E-06	30
**Liver Necrosis/Cell Death**	5,50E-01 - 2,26E-05	19
**Liver Fibrosis**	2,23E-01 - 2,95E-04	25

Nephrotoxicity

(c) 2000-2023 QIAGEN. All rights reserved.

INGENUITY PATHWAY ANALYSIS

Name	p-value range	# Molecules
**Renal Proliferation**	3,25E-01 - 3,73E-04	17
**Renal Damage**	5,31E-01 - 2,19E-03	17
**Renal Necrosis/Cell Death**	1,00E00 - 3,09E-03	24
**Renal Inflammation**	1,00E00 - 1,74E-02	15
**Renal Nephritis**	1,00E00 - 1,74E-02	15

Top Regulator Effect Networks

ID	Regulators	Disease & Functions	Consistency Score
**1**	ABCG1,ACACB,ACSS2,C4BP,CASP1,CYP27A1,CYP51A1 (+19 more)	Cell proliferation of tumor cell lines (+4 more)	65,324
**2**	ACACB,AIM2,CSF1,CYP27A1,CYP51A1,CYP7A1,DBI,INSIG1 (+8 more)	Cell cycle progression (+4 more)	35,921
**3**	ABCG1,ACACB,ACSS2,C4BP,CASP1,CTBP2,CYP27A1,CYP46A1 (+29 more)	Cell death of tumor cell lines (+8 more)	30,815
**4**	ACACB,AIM2,CKAP2L,CSF1,CYP27A1,CYP51A1,CYP7A1,DBI (+15 more)	Cell proliferation of tumor cell lines (+5 more)	24,11
**5**	ACTB,CKAP2L,CSF1,CYP27A1,CYP7A1,EP400,H2AZ1,INSIG1 (+11 more)	Cell proliferation of tumor cell lines (+6 more)	22,112

Top Networks

ID	Associated Network Functions	Score

(c) 2000-2023 QIAGEN. All rights reserved.

INGENUITY PATHWAY ANALYSIS

**1**	Cell Cycle, Cellular Assembly and Organization, DNA Replication, Recombination, and Repair	51
**2**	Cellular Assembly and Organization, Cellular Function and Maintenance, Cell Cycle	46
**3**	Metabolic Disease, Organismal Injury and Abnormalities, DNA Replication, Recombination, and Repair	43
**4**	Cell Cycle, Cellular Movement, Cellular Assembly and Organization	43
**5**	Cell Cycle, Cellular Assembly and Organization, DNA Replication, Recombination, and Repair	41

Top Tox Lists

Name	p-value	Overlap
**Cell Cycle: G2/M DNA Damage Checkpoint Regulation**	7,90E-09	21,2 % 11/52
**Cholesterol Biosynthesis**	5,27E-07	37,5 % 6/16
**Liver Necrosis/Cell Death**	1,46E-04	5,4 % 19/349

(c) 2000-2023 QIAGEN. All rights reserved.

INGENUITY PATHWAY ANALYSIS

**LXR/RXR Activation**	2,55E-04	8,1 %	10/123
**Hepatic Fibrosis**	3,04E-04	5,3 %	18/340

Top My Lists

Top My Pathways

Top Analysis-Ready Molecules

Expr Log Ratio

Molecules	Expr. Value	Chart
**RNF112**	↑ 3,607	
**CORO2B**	↑ 2,833	
**PRUNE2**	↑ 2,680	
**ACSM2B**	↑ 2,152	
**ACSM2A**	↑ 1,911	
**ANGPTL4**	↑ 1,841	
**SBSN**	↑ 1,792	
**CYP2E1**	↑ 1,689	
**STUM**	↑ 1,681	
**PRDM6**	↑ 1,508	

Expr Log Ratio

(c) 2000-2023 QIAGEN. All rights reserved.

INGENUITY PATHWAY ANALYSIS

Molecules	Expr. Value	Chart
CDA	↓ -3,480	
SERPING1	↓ -1,960	
TRPV2	↓ -1,560	
TOGARAM2	↓ -1,375	
IQGAP2	↓ -1,373	
PDE3A	↓ -1,277	
BMP3	↓ -1,270	
MKI67	↓ -1,214	
KIF14	↓ -1,177	
NCAPG	↓ -1,174	

(c) 2000-2023 QIAGEN. All rights reserved.

INGENUITY PATHWAY ANALYSIS

INGENUITY
PATHWAY ANALYSIS

Analysis Name: SA1A - Monoculture - CCDSK (with homologs) - 2023-02-16 11:16 vorm.
Analysis Creation Date: 2023-02-16
Build version: exported
Content version: 84978992 (Release Date: 2022-11-27)

Experiment Metadata

Name	Value

Analysis Settings

Reference set: Ingenuity Knowledge Base (Genes Only)
Relationship to include: Direct and Indirect
Includes Endogenous Chemicals
Optional Analyses: My Pathways My List

Filter Summary:
Consider only molecules and/or relationships where
(species = Uncategorized OR Human OR Mouse OR Rat) AND
(confidence = Experimentally Observed) AND
(tissues/cell lines = Other Dendritic cells OR Corpus Callosum OR Pancreas OR Other Melanoma Cell Lines OR Other Cell Line OR Hematopoietic progenitor cells OR Murine NKT cells OR SN12C OR U251 OR Central memory helper T cells OR Stromal cells OR White Matter OR Neutrophils OR Placenta OR Other Lymphoma Cell Lines OR Cornea OR NCI-H23 OR Other Smooth muscle cells OR Cos-7 cells OR SF-

(c) 2000-2023 QIAGEN. All rights reserved.

268 OR CD34+ cells OR Th2 cells OR Vd2 Gamma-delta T cells OR Memory T lymphocytes not otherwise specified OR Chondrocytes OR Skeletal Muscle OR Striatum OR HT29 OR Central memory cytotoxic T cells OR Trigeminal Ganglion OR J-774A.1 OR A549-ATCC OR Nucleus Accumbens OR Plasma cells OR A375 OR Granule cells OR SK-MEL-2 OR HepG2 OR Forestomach OR Amygdala OR Cardiomyocytes OR Granulocytes not otherwise specified OR SF-295 OR CD56dim NK cells OR Spleen OR RBL-2H3 OR CAKI-1 OR MCF7 OR Activated helper T cells OR Granulosa cells OR Activated Vd1 Gamma-delta T cells OR T lymphocytes not otherwise specified OR Colon Cancer Cell Lines not otherwise specified OR Other Breast Cancer Cell Lines OR Brainstem OR Cells not otherwise specified OR Peripheral blood monocytes OR Other Monocyte-derived dendritic cells OR NB4 OR Medulla Oblongata OR WEHI-231 OR Cytotoxic T cells OR BT-549 OR Lymphoma Cell Lines not otherwise specified OR Beta islet cells OR NCI-H332M OR Other Colon Cancer Cell Lines OR Other Neuroblastoma Cell Lines OR Other Stem cells OR Caudate Nucleus OR Melanocytes OR Other Cells OR Other Ovarian Cancer Cell Lines OR Monocyte-derived dendritic cells not otherwise specified OR Other Organ Systems OR Peripheral blood lymphocytes OR Other Hepatoma Cell Lines OR 3T3-L1 cells OR Other Bone marrow cells OR Fibroblasts OR HOP-92 OR Salivary Gland OR Large Intestine OR CNS Cell Lines not otherwise specified OR Bone marrow-derived macrophages OR Effector memory cytotoxic T cells OR Naive helper T cells OR UACC-62 OR OVCAR-8 OR OVCAR-4 OR MEF cells OR SK-MEL-5 OR Microvascular endothelial cells OR Other Prostate Cancer Cell Lines OR 786-0 OR Granule Cell Layer OR Lymphocytes not otherwise specified OR NCI-ADR-RES OR Dendritic cells not otherwise specified OR Stomach OR CD56bright NK cells OR Other Kidney cell lines OR Min6 OR Purkinje cells OR Activated CD56bright NK cells OR Brain OR Langerhans cells OR Mesenchymal stem cells OR Thalamus OR J774 OR Other CNS Cell Lines OR MDA-MB-468 OR UACC-257 OR Bone marrow cells not otherwise specified OR Other Nervous System OR MDA-MB-435 OR Jurkat OR RKO OR Macrophage Cancer Cell Lines not otherwise specified OR Cell Line not otherwise specified OR U266 OR Other Memory T lymphocytes OR Skin OR Monocyte-derived macrophage OR ACHN OR Other Granulocytes OR Melanoma Cell Lines not otherwise specified OR HCT-15 OR Pheochromocytoma cell lines not otherwise specified OR MOLT-4 OR RAW 264.7 OR Lung Cancer Cell Lines not otherwise specified OR SK-N-SH OR Other Pancreatic Cancer Cell Lines OR COLO205 OR Epithelial cells not otherwise specified OR Pro-B lymphocytes OR SK-OV-3 OR Testis OR Other Neurons OR RXF-393 OR Gray Matter OR Other NK cells OR BT-474 OR Olfactory Bulb OR Prostate Gland OR P19 OR Vd1 Gamma-delta T cells OR A2780 OR HS 578T OR Cortical neurons OR Other Osteosarcoma Cell Lines OR Megakaryocytes OR Memory B cells OR Kidney OR Trachea OR U937 OR Breast Cancer Cell Lines not otherwise specified OR LOX IMVI OR RPMI-8266 OR Natural T-regulatory cells OR MG-63 OR Th17 cells OR Oocytes OR Esophagus OR Other Endothelial cells OR TK-10 OR Monocytes not otherwise specified OR 293 cells OR SNB-75 OR Caco2 cells OR EKVX OR Other Lymphocytes OR Peritoneal macrophages OR Calvaria OR Other Immune cell lines OR Other Macrophage Cancer Cell Lines OR Hep3B OR Cervical cancer cell line not otherwise specified OR Prostate Cancer Cell Lines not otherwise specified OR T47-D OR Mammary Gland OR CD4+ T-lymphocytes OR Cerebellum OR Leukemia Cell Lines not otherwise specified OR CCRF-CEM OR KM-12 OR BDCA-1+ dendritic cells OR Hepatoma Cell Lines not otherwise specified OR Other Peripheral blood leukocytes OR U87MG OR Sciatic Nerve OR Other Macrophages

(c) 2000-2023 QIAGEN. All rights reserved.

INGENUITY PATHWAY ANALYSIS

OR Immune cell lines not otherwise specified OR LNCaP cells OR MDA-MB-231 OR HeLa OR HMC-1 OR UO-31 OR Crypt OR Activated Vd2 Gamma-delta T cells OR Naive B cells OR Parietal Lobe OR Dermis OR Thyroid Gland OR Other Pheochromocytoma cell lines OR NIH/3T3 cells OR Other T lymphocytes OR Cartilage Tissue OR Other Kidney Cancer Cell Lines OR PBMCs OR Adrenal Gland OR Effector memory RA+ cytotoxic T cells OR Heart OR Smooth Muscle OR Liver OR Osteoblasts OR Bladder OR Epidermis OR Mast cells OR Small Intestine OR Osteosarcoma Cell Lines not otherwise specified OR A498 OR Other Monocytes OR Peripheral blood leukocytes not otherwise specified OR Pituitary Gland OR Blood platelets OR THP-1 OR Macrophages not otherwise specified OR Myeloid dendritic cells OR Dorsal Root Ganglion OR HCC-2998 OR Adipose OR Cerebral Ventricles OR Pyramidal neurons OR NCI-H226 OR U2OS OR Myeloma Cell Lines not otherwise specified OR Substantia Nigra OR Cerebral Cortex OR Other Myeloma Cell Lines OR M14 OR Thymus OR DU-145 OR HL-60 OR Astrocytes OR B lymphocytes not otherwise specified OR Uterus OR PC-12 cells OR Vascular smooth muscle cells OR Th1 cells OR HuH7 OR Microglia OR Choroid Plexus OR Other Immune cells OR Spinal Cord OR Mature monocyte-derived dendritic cells OR Fibroblast cell lines not otherwise specified OR HCT-116 OR NT2/D1 OR Intraepithelial T lymphocytes OR Lung OR NCI-H522 OR PANC-1 OR Tissues and Primary Cells not otherwise specified OR Other Mononuclear leukocytes OR SW-480 OR BDCA-3+ dendritic cells OR Smooth muscle cells not otherwise specified OR SK-MEL-28 OR BA/F3 OR Ovarian Cancer Cell Lines not otherwise specified OR Other Epithelial cells OR Putamen OR Neurons not otherwise specified OR Effector T cells OR Other Tissues and Primary Cells OR Neuroblastoma Cell Lines not otherwise specified OR Pancreatic Cancer Cell Lines not otherwise specified OR MDA-MB-361 OR SR OR Bone marrow-derived dendritic cells OR Lens OR MALME-3M OR HOP-62 OR HEL OR Activated CD56dim NK cells OR Nervous System not otherwise specified OR Plasmacytoid dendritic cells OR Swiss 3T3 cells OR Endothelial cells not otherwise specified OR Hepatocytes OR Ventricular Zone OR PC-3 OR Immature monocyte-derived dendritic cells OR Immune cells not otherwise specified OR Retina OR Mononuclear leukocytes not otherwise specified OR Organ Systems not otherwise specified OR Other Lung Cancer Cell Lines OR Subventricular Zone OR H460 OR K-562 OR INS-1 OR Kidney cell lines not otherwise specified OR Hippocampus OR Teratocarcinoma Cell Lines not otherwise specified OR Sertoli cells OR Kidney Cancer Cell Lines not otherwise specified OR Effector memory helper T cells OR SF-539 OR Adipocytes OR MDA-N OR Eosinophils OR NK cells not otherwise specified OR Splenocytes OR OVCAR-3 OR SW-620 OR Ovary OR Embryonic stem cells OR HUVEC cells OR OVCAR-5 OR Hypothalamus OR Stem cells not otherwise specified OR Other Leukemia Cell Lines OR Other Teratocarcinoma Cell Lines OR Thymocytes OR Other Cervical cancer cell line OR Keratinocytes OR Pre-B lymphocytes OR Other B lymphocytes OR Other Fibroblast cell lines OR Lymph node OR IGROV1) AND
(mol. types = biologic drug OR canonical pathway OR chemical - endogenous mammalian OR chemical - endogenous non-mammalian OR chemical - kinase inhibitor OR chemical - other OR chemical - protease inhibitor OR chemical drug OR chemical reagent OR chemical toxicant OR complex OR cytokine OR disease OR enzyme OR function OR fusion gene/product OR G-protein coupled receptor OR group OR growth factor OR ion channel OR kinase OR ligand-dependent nuclear receptor OR mature microRNA OR microRNA OR other OR peptidase OR phosphatase OR transcription regulator OR translation regulator OR transmembrane receptor OR transporter) AND

(c) 2000-2023 QIAGEN. All rights reserved.

INGENUITY PATHWAY ANALYSIS

(data sources = An Open Access Database of Genome-wide Association Results OR BIND OR BioGRID OR Catalogue Of Somatic Mutations In Cancer (COSMIC) OR Chemical Carcinogenesis Research Information System (CCRIS) OR Clinical Genome Resource (ClinGen) OR ClinicalTrials.gov OR ClinVar OR Cognia OR DIP OR DrugBank OR Gene Ontology (GO) OR GVK Biosciences OR Hazardous Substances Data Bank (HSDB) OR HumanCyc OR Ingenuity Expert Findings OR Ingenuity ExpertAssist Findings OR IntAct OR Interactome studies OR MIPS OR miRBase OR miRecords OR Mouse Genome Database (MGD) OR Obesity Gene Map Database OR Online Mendelian Inheritance in Man (OMIM) OR TarBase OR TargetScan Human OR TargetScan Mouse)

Top Canonical Pathways

Name	p-value	Overlap
**Axonal Guidance Signaling**	9,50E-10	9,0 % 46/509
**Superpathway of Cholesterol Biosynthesis**	1,74E-08	34,5 % 10/29
**Actin Cytoskeleton Signaling**	4,99E-08	11,1 % 27/244
**Cardiac Hypertrophy Signaling (Enhanced)**	1,46E-07	7,9 % 43/542
**ILK Signaling**	2,65E-07	11,4 % 23/201

Top Upstream Regulators

Upstream Regulators

Name	p-value	Predicted Activation
**HRAS**	1,23E-26	Inhibited
**TGFB1**	2,20E-25	
**beta-estradiol**	3,09E-25	
**CG**	3,27E-18	

(c) 2000-2023 QIAGEN. All rights reserved.

INGENUITY PATHWAY ANALYSIS

**TNF**	1,28E-17

Causal Network

Name	p-value	Predicted Activation
**LIN28A**	1,76E-27	
**8-bromo-cAMP**	1,20E-26	
**medroxyprogesterone acetate**	1,37E-26	
**NCOA3**	9,12E-26	
**lapatinib**	9,74E-26	

Top Diseases and Bio Functions

Diseases and Disorders

Name	p-value range	# Molecules
**Cancer**	3,17E-06 - 2,41E-58	773
**Organismal Injury and Abnormalities**	3,17E-06 - 2,41E-58	782
**Endocrine System Disorders**	2,77E-06 - 9,28E-44	689
**Gastrointestinal Disease**	3,17E-06 - 2,91E-42	716
**Reproductive System Disease**	2,12E-06 - 1,16E-30	617

Molecular and Cellular Functions

(c) 2000-2023 QIAGEN. All rights reserved.

INGENUITY PATHWAY ANALYSIS

Name	p-value range	# Molecules
Cellular Movement	3,17E-06 - 4,62E-15	240
Cellular Assembly and Organization	2,59E-06 - 8,02E-14	247
Cellular Function and Maintenance	2,59E-06 - 8,02E-14	205
Cellular Development	2,92E-06 - 1,20E-11	306
Cellular Growth and Proliferation	2,92E-06 - 1,20E-11	297

Physiological System Development and Function

Name	p-value range	# Molecules
Cardiovascular System Development and Function	2,57E-06 - 2,02E-20	197
Organismal Development	2,59E-06 - 2,02E-20	297
Embryonic Development	3,00E-06 - 6,33E-15	162
Tissue Development	2,92E-06 - 4,15E-14	318
Organismal Survival	3,76E-13 - 3,76E-13	229

Top Tox Functions

Assays: Clinical Chemistry and Hematology

Name	p-value range	# Molecules
Increased Levels of Red Blood Cells	1,80E-03 - 1,80E-03	11
Increased Levels of Alkaline Phosphatase	3,32E-02 - 2,39E-03	9

(c) 2000-2023 QIAGEN. All rights reserved.

Decreased Levels of Albumin	2,37E-01 - 8,08E-03	5
Increased Levels of Albumin	3,32E-02 - 3,32E-02	1
Decreased Levels of Hematocrit	4,15E-02 - 4,15E-02	2

Cardiotoxicity

Name	p-value range	# Molecules
Cardiac Enlargement	1,00E00 - 3,62E-09	67
Cardiac Arteriopathy	2,62E-01 - 3,95E-09	41
Heart Failure	2,62E-01 - 5,44E-07	35
Cardiac Dysfunction	4,74E-01 - 1,97E-06	34
Cardiac Arrythmia	1,00E00 - 6,91E-06	31

Hepatotoxicity

Name	p-value range	# Molecules
Liver Hyperplasia/Hyperproliferation	1,00E00 - 1,82E-14	378
Liver Damage	1,26E-01 - 3,17E-06	26
Liver Proliferation	2,26E-01 - 1,66E-05	24
Liver Necrosis/Cell Death	5,08E-01 - 1,91E-05	25
Liver Steatosis	1,00E00 - 5,39E-05	39

Nephrotoxicity

(c) 2000-2023 QIAGEN. All rights reserved.

INGENUITY PATHWAY ANALYSIS

Name	p-value range	# Molecules
**Kidney Failure**	4,56E-01 - 4,66E-05	27
**Glomerular Injury**	6,37E-01 - 3,15E-04	43
**Renal Inflammation**	4,18E-01 - 3,15E-04	30
**Renal Nephritis**	4,18E-01 - 3,15E-04	30
**Renal Necrosis/Cell Death**	5,40E-01 - 5,88E-04	36

Top Regulator Effect Networks

ID	Regulators	Disease & Functions	Consistency Score
**1**	CD40,CD40LG,CIC,Collagen type I (complex) (+6 more)	Congenital malformation of genitourinary system (+3 more)	15,087
**2**	CD3E,CSF1R,Mapk,SRC	Differentiation of bone (+3 more)	14,067
**3**	BCL6,C4BP,CYP7A1,EIF6,ELOVL3,FGF21,Gm35986,ILF3 (+10 more)	Fatty acid metabolism,Metabolism of cholesterol	10,028
**4**	CD40LG,CSF1R,EIF2AK3,F3,KL,PTX3	Differentiation of bone,Invasion of cells (+1 more)	9,664
**5**	CFTR,CYP51A1,CYP7A1,HMG20A,INSIG2,MAP2K5,MAPK7 (+15 more)	Metabolism of cholesterol	9,216

Top Networks

ID	Associated Network Functions	Score

(c) 2000-2023 QIAGEN. All rights reserved.

INGENUITY PATHWAY ANALYSIS

**1**	RNA Post-Transcriptional Modification, Cellular Assembly and Organization, Cellular Function and Maintenance	52
**2**	Cell Morphology, Cellular Development, Cellular Growth and Proliferation	47
**3**	Cellular Movement, Nervous System Development and Function, Carbohydrate Metabolism	44
**4**	Post-Translational Modification, Lipid Metabolism, Molecular Transport	42
**5**	Hereditary Disorder, Metabolic Disease, Organismal Injury and Abnormalities	40

Top Tox Lists

Name	p-value	Overlap
**Cholesterol Biosynthesis**	1,45E-08	50,0 % 8/16
**Cardiac Hypertrophy**	7,56E-07	8,6 % 33/385
**Cardiac Fibrosis**	6,60E-06	8,5 % 28/331
**Cardiac Necrosis/Cell Death**	8,27E-06	8,4 % 28/335
**Liver Proliferation**	2,53E-05	8,5 % 24/281

(c) 2000-2023 QIAGEN. All rights reserved.

Top My Lists

Top My Pathways

Top Analysis-Ready Molecules

Expr Log Ratio

Molecules	Expr. Value	Chart
ASB2	↑ 3,421	
SERPINB7	↑ 2,369	
SLC47A1	↑ 2,093	
ITGA8	↑ 1,602	
SCN3A	↑ 1,573	
GNA14	↑ 1,545	
FBXL22	↑ 1,448	
PROC	↑ 1,366	
IGFBPL1	↑ 1,364	
KLHL38	↑ 1,363	

Expr Log Ratio

(c) 2000-2023 QIAGEN. All rights reserved.

INGENUITY PATHWAY ANALYSIS

Molecules	Expr. Value	Chart
**PRODH2**	↓ -4,976	
**SHH**	↓ -3,884	
**ANKRD34B**	↓ -3,556	
**VWA2**	↓ -3,352	
**RIMKLA**	↓ -3,121	
**RBM11**	↓ -2,982	
**MMP9**	↓ -2,870	
**SLC17A1**	↓ -2,786	
**HSD11B2**	↓ -2,634	
**DMBX1**	↓ -2,486	

(c) 2000-2023 QIAGEN. All rights reserved.

INGENUITY PATHWAY ANALYSIS

Analysis Name: SA1A - Monoculture - HAoSMC (with homologs) - 2023-02-16 11:17 vorm.
Analysis Creation Date: 2023-02-16
Build version: exported
Content version: 84978992 (Release Date: 2022-11-27)

Experiment Metadata

Name	Value

Analysis Settings

Reference set: Ingenuity Knowledge Base (Genes Only)
Relationship to include: Direct and Indirect
Includes Endogenous Chemicals
Optional Analyses: My Pathways My List

Filter Summary:
Consider only molecules and/or relationships where
(species = Uncategorized OR Human OR Mouse OR Rat) AND
(confidence = Experimentally Observed) AND
(tissues/cell lines = Other Dendritic cells OR Corpus Callosum OR Pancreas OR Other Melanoma Cell Lines OR Other Cell Line OR Hematopoietic progenitor cells OR Murine NKT cells OR SN12C OR U251 OR Central memory helper T cells OR Stromal cells OR White Matter OR Neutrophils OR Placenta OR Other Lymphoma Cell Lines OR Cornea OR NCI-H23 OR Other Smooth muscle cells OR Cos-7 cells OR SF-

(c) 2000-2023 QIAGEN. All rights reserved.

268 OR CD34+ cells OR Th2 cells OR Vd2 Gamma-delta T cells OR Memory T lymphocytes not otherwise specified OR Chondrocytes OR Skeletal Muscle OR Striatum OR HT29 OR Central memory cytotoxic T cells OR Trigeminal Ganglion OR J-774A.1 OR A549-ATCC OR Nucleus Accumbens OR Plasma cells OR A375 OR Granule cells OR SK-MEL-2 OR HepG2 OR Forestomach OR Amygdala OR Cardiomyocytes OR Granulocytes not otherwise specified OR SF-295 OR CD56dim NK cells OR Spleen OR RBL-2H3 OR CAKI-1 OR MCF7 OR Activated helper T cells OR Granulosa cells OR Activated Vd1 Gamma-delta T cells OR T lymphocytes not otherwise specified OR Colon Cancer Cell Lines not otherwise specified OR Other Breast Cancer Cell Lines OR Brainstem OR Cells not otherwise specified OR Peripheral blood monocytes OR Other Monocyte-derived dendritic cells OR NB4 OR Medulla Oblongata OR WEHI-231 OR Cytotoxic T cells OR BT-549 OR Lymphoma Cell Lines not otherwise specified OR Beta islet cells OR NCI-H332M OR Other Colon Cancer Cell Lines OR Other Neuroblastoma Cell Lines OR Other Stem cells OR Caudate Nucleus OR Melanocytes OR Other Cells OR Other Ovarian Cancer Cell Lines OR Monocyte-derived dendritic cells not otherwise specified OR Other Organ Systems OR Peripheral blood lymphocytes OR Other Hepatoma Cell Lines OR 3T3-L1 cells OR Other Bone marrow cells OR Fibroblasts OR HOP-92 OR Salivary Gland OR Large Intestine OR CNS Cell Lines not otherwise specified OR Bone marrow-derived macrophages OR Effector memory cytotoxic T cells OR Naive helper T cells OR UACC-62 OR OVCAR-8 OR OVCAR-4 OR MEF cells OR SK-MEL-5 OR Microvascular endothelial cells OR Other Prostate Cancer Cell Lines OR 786-0 OR Granule Cell Layer OR Lymphocytes not otherwise specified OR NCI-ADR-RES OR Dendritic cells not otherwise specified OR Stomach OR CD56bright NK cells OR Other Kidney cell lines OR Min6 OR Purkinje cells OR Activated CD56bright NK cells OR Brain OR Langerhans cells OR Mesenchymal stem cells OR Thalamus OR J774 OR Other CNS Cell Lines OR MDA-MB-468 OR UACC-257 OR Bone marrow cells not otherwise specified OR Other Nervous System OR MDA-MB-435 OR Jurkat OR RKO OR Macrophage Cancer Cell Lines not otherwise specified OR Cell Line not otherwise specified OR U266 OR Other Memory T lymphocytes OR Skin OR Monocyte-derived macrophage OR ACHN OR Other Granulocytes OR Melanoma Cell Lines not otherwise specified OR HCT-15 OR Pheochromocytoma cell lines not otherwise specified OR MOLT-4 OR RAW 264.7 OR Lung Cancer Cell Lines not otherwise specified OR SK-N-SH OR Other Pancreatic Cancer Cell Lines OR COLO205 OR Epithelial cells not otherwise specified OR Pro-B lymphocytes OR SK-OV-3 OR Testis OR Other Neurons OR RXF-393 OR Gray Matter OR Other NK cells OR BT-474 OR Olfactory Bulb OR Prostate Gland OR P19 OR Vd1 Gamma-delta T cells OR A2780 OR HS 578T OR Cortical neurons OR Other Osteosarcoma Cell Lines OR Megakaryocytes OR Memory B cells OR Kidney OR Trachea OR U937 OR Breast Cancer Cell Lines not otherwise specified OR LOX IMVI OR RPMI-8266 OR Natural T-regulatory cells OR MG-63 OR Th17 cells OR Oocytes OR Esophagus OR Other Endothelial cells OR TK-10 OR Monocytes not otherwise specified OR 293 cells OR SNB-75 OR Caco2 cells OR EKVX OR Other Lymphocytes OR Peritoneal macrophages OR Calvaria OR Other Immune cell lines OR Other Macrophage Cancer Cell Lines OR Hep3B OR Cervical cancer cell line not otherwise specified OR Prostate Cancer Cell Lines not otherwise specified OR T47-D OR Mammary Gland OR CD4+ T-lymphocytes OR Cerebellum OR Leukemia Cell Lines not otherwise specified OR CCRF-CEM OR KM-12 OR BDCA-1+ dendritic cells OR Hepatoma Cell Lines not otherwise specified OR Other Peripheral blood leukocytes OR U87MG OR Sciatic Nerve OR Other Macrophages

(c) 2000-2023 QIAGEN. All rights reserved.

INGENUITY PATHWAY ANALYSIS

OR Immune cell lines not otherwise specified OR LNCaP cells OR MDA-MB-231 OR HeLa OR HMC-1 OR UO-31 OR Crypt OR Activated Vd2 Gamma-delta T cells OR Naive B cells OR Parietal Lobe OR Dermis OR Thyroid Gland OR Other Pheochromocytoma cell lines OR NIH/3T3 cells OR Other T lymphocytes OR Cartilage Tissue OR Other Kidney Cancer Cell Lines OR PBMCs OR Adrenal Gland OR Effector memory RA+ cytotoxic T cells OR Heart OR Smooth Muscle OR Liver OR Osteoblasts OR Bladder OR Epidermis OR Mast cells OR Small Intestine OR Osteosarcoma Cell Lines not otherwise specified OR A498 OR Other Monocytes OR Peripheral blood leukocytes not otherwise specified OR Pituitary Gland OR Blood platelets OR THP-1 OR Macrophages not otherwise specified OR Myeloid dendritic cells OR Dorsal Root Ganglion OR HCC-2998 OR Adipose OR Cerebral Ventricles OR Pyramidal neurons OR NCI-H226 OR U2OS OR Myeloma Cell Lines not otherwise specified OR Substantia Nigra OR Cerebral Cortex OR Other Myeloma Cell Lines OR M14 OR Thymus OR DU-145 OR HL-60 OR Astrocytes OR B lymphocytes not otherwise specified OR Uterus OR PC-12 cells OR Vascular smooth muscle cells OR Th1 cells OR HuH7 OR Microglia OR Choroid Plexus OR Other Immune cells OR Spinal Cord OR Mature monocyte-derived dendritic cells OR Fibroblast cell lines not otherwise specified OR HCT-116 OR NT2/D1 OR Intraepithelial T lymphocytes OR Lung OR NCI-H522 OR PANC-1 OR Tissues and Primary Cells not otherwise specified OR Other Mononuclear leukocytes OR SW-480 OR BDCA-3+ dendritic cells OR Smooth muscle cells not otherwise specified OR SK-MEL-28 OR BA/F3 OR Ovarian Cancer Cell Lines not otherwise specified OR Other Epithelial cells OR Putamen OR Neurons not otherwise specified OR Effector T cells OR Other Tissues and Primary Cells OR Neuroblastoma Cell Lines not otherwise specified OR Pancreatic Cancer Cell Lines not otherwise specified OR MDA-MB-361 OR SR OR Bone marrow-derived dendritic cells OR Lens OR MALME-3M OR HOP-62 OR HEL OR Activated CD56dim NK cells OR Nervous System not otherwise specified OR Plasmacytoid dendritic cells OR Swiss 3T3 cells OR Endothelial cells not otherwise specified OR Hepatocytes OR Ventricular Zone OR PC-3 OR Immature monocyte-derived dendritic cells OR Immune cells not otherwise specified OR Retina OR Mononuclear leukocytes not otherwise specified OR Organ Systems not otherwise specified OR Other Lung Cancer Cell Lines OR Subventricular Zone OR H460 OR K-562 OR INS-1 OR Kidney cell lines not otherwise specified OR Hippocampus OR Teratocarcinoma Cell Lines not otherwise specified OR Sertoli cells OR Kidney Cancer Cell Lines not otherwise specified OR Effector memory helper T cells OR SF-539 OR Adipocytes OR MDA-N OR Eosinophils OR NK cells not otherwise specified OR Splenocytes OR OVCAR-3 OR SW-620 OR Ovary OR Embryonic stem cells OR HUVEC cells OR OVCAR-5 OR Hypothalamus OR Stem cells not otherwise specified OR Other Leukemia Cell Lines OR Other Teratocarcinoma Cell Lines OR Thymocytes OR Other Cervical cancer cell line OR Keratinocytes OR Pre-B lymphocytes OR Other B lymphocytes OR Other Fibroblast cell lines OR Lymph node OR IGROV1) AND
(mol. types = biologic drug OR canonical pathway OR chemical - endogenous mammalian OR chemical - endogenous non-mammalian OR chemical - kinase inhibitor OR chemical - other OR chemical - protease inhibitor OR chemical drug OR chemical reagent OR chemical toxicant OR complex OR cytokine OR disease OR enzyme OR function OR fusion gene/product OR G-protein coupled receptor OR group OR growth factor OR ion channel OR kinase OR ligand-dependent nuclear receptor OR mature microRNA OR microRNA OR other OR peptidase OR phosphatase OR transcription regulator OR translation regulator OR transmembrane receptor OR transporter) AND

(c) 2000-2023 QIAGEN. All rights reserved.

(data sources = An Open Access Database of Genome-wide Association Results OR BIND OR BioGRID OR Catalogue Of Somatic Mutations In Cancer (COSMIC) OR Chemical Carcinogenesis Research Information System (CCRIS) OR Clinical Genome Resource (ClinGen) OR ClinicalTrials.gov OR ClinVar OR Cognia OR DIP OR DrugBank OR Gene Ontology (GO) OR GVK Biosciences OR Hazardous Substances Data Bank (HSDB) OR HumanCyc OR Ingenuity Expert Findings OR Ingenuity ExpertAssist Findings OR IntAct OR Interactome studies OR MIPS OR miRBase OR miRecords OR Mouse Genome Database (MGD) OR Obesity Gene Map Database OR Online Mendelian Inheritance in Man (OMIM) OR TarBase OR TargetScan Human OR TargetScan Mouse)

Top Canonical Pathways

Name	p-value	Overlap
**Adipogenesis pathway**	9,55E-11	27,3 % 38/139
**Pulmonary Fibrosis Idiopathic Signaling Pathway**	1,03E-07	17,8 % 58/326
**Unfolded protein response**	1,06E-07	27,8 % 25/90
**Molecular Mechanisms of Cancer**	5,39E-07	15,8 % 71/450
**Cardiac Hypertrophy Signaling (Enhanced)**	1,65E-06	14,8 % 80/542

Top Upstream Regulators

Upstream Regulators

Name	p-value	Predicted Activation
**beta-estradiol**	1,08E-36	Activated
**TGFB1**	6,08E-30	Activated
**ESR2**	2,72E-28	

(c) 2000-2023 QIAGEN. All rights reserved.

INGENUITY PATHWAY ANALYSIS

**dexamethasone**	3,69E-26	Activated
**TNF**	2,76E-24	

Causal Network

Name	p-value	Predicted Activation
**beta-estradiol**	9,77E-40	Activated
**RANBP1**	7,97E-36	Activated
**PSMC5**	1,12E-35	Inhibited
**CG**	1,96E-35	Activated
**CDK9**	2,15E-35	

Top Diseases and Bio Functions

Diseases and Disorders

Name	p-value range	# Molecules
**Cancer**	1,74E-08 - 2,24E-151	2015
**Organismal Injury and Abnormalities**	2,25E-08 - 2,24E-151	2038
**Endocrine System Disorders**	1,74E-08 - 1,41E-101	1813
**Gastrointestinal Disease**	1,60E-08 - 1,04E-76	1821
**Reproductive System Disease**	4,96E-09 - 5,23E-52	1499

Molecular and Cellular Functions

(c) 2000-2023 QIAGEN. All rights reserved.

INGENUITY PATHWAY ANALYSIS

Name	p-value range	# Molecules
**Molecular Transport**	7,73E-11 - 1,40E-24	479
**Cellular Movement**	2,00E-08 - 2,98E-24	586
**Cell Death and Survival**	1,91E-08 - 6,94E-24	753
**Cellular Assembly and Organization**	2,80E-09 - 3,43E-19	434
**Cellular Function and Maintenance**	2,05E-08 - 3,43E-19	691

Physiological System Development and Function

Name	p-value range	# Molecules
**Organismal Development**	2,01E-08 - 1,05E-25	820
**Cardiovascular System Development and Function**	2,00E-08 - 2,70E-24	409
**Organismal Survival**	4,69E-12 - 1,20E-22	599
**Embryonic Development**	7,90E-09 - 4,88E-20	488
**Tissue Development**	2,29E-08 - 5,94E-17	645

Top Tox Functions

Assays: Clinical Chemistry and Hematology

Name	p-value range	# Molecules
**Increased Levels of Red Blood Cells**	5,53E-05 - 5,53E-05	24
**Increased Levels of Alkaline Phosphatase**	1,14E-03 - 1,06E-03	17

(c) 2000-2023 QIAGEN. All rights reserved.

**Increased Levels of LDH**	2,00E-01 - 3,58E-03	9
**Increased Levels of Hematocrit**	5,83E-03 - 5,83E-03	17
**Increased Levels of Creatinine**	1,00E00 - 2,81E-02	11

Cardiotoxicity

Name	p-value range	# Molecules
**Cardiac Necrosis/Cell Death**	4,19E-01 - 1,05E-06	57
**Cardiac Arteriopathy**	5,96E-01 - 1,25E-06	69
**Cardiac Enlargement**	1,00E00 - 6,66E-06	119
**Congenital Heart Anomaly**	1,00E00 - 3,36E-05	62
**Cardiac Fibrosis**	5,96E-01 - 5,09E-05	52

Hepatotoxicity

Name	p-value range	# Molecules
**Liver Hyperplasia/Hyperproliferation**	1,00E00 - 1,61E-29	963
**Liver Steatosis**	3,64E-01 - 2,13E-11	102
**Liver Necrosis/Cell Death**	2,72E-01 - 6,17E-09	61
**Liver Proliferation**	2,38E-01 - 1,27E-06	53
**Hepatocellular carcinoma**	1,00E00 - 1,00E-04	259

Nephrotoxicity

(c) 2000-2023 QIAGEN. All rights reserved.

INGENUITY PATHWAY ANALYSIS

Name	p-value range	# Molecules
**Renal Necrosis/Cell Death**	5,15E-01 - 2,58E-08	107
**Renal Damage**	1,00E00 - 6,78E-05	52
**Glomerular Injury**	1,00E00 - 2,30E-04	92
**Renal Fibrosis**	4,69E-01 - 2,30E-04	29
**Renal Inflammation**	1,00E00 - 2,50E-04	59

Top Regulator Effect Networks

ID	Regulators	Disease & Functions	Consistency Score
**1**	CDKN1A,CXCL12,EDN1,EIF4EBP1,F2,HIF1A,IL13,INSR (+7 more)	Proliferation of hepatic stellate cells	14,0
**2**	KDM3A,MIR31HG	Cell movement of leukocytes (+2 more)	6,957
**3**	CYP19A1,FSH,G protein alpha i,INSIG1,MFSD2A (+6 more)	Metabolism of prostaglandin	5,59
**4**	F2RL1,growth factor	Activation of cells,Failure of kidney (+3 more)	5,5
**5**	PRRX1	Activation of cells,Cell movement	4,491

Top Networks

ID	Associated Network Functions	Score
**1**	Amino Acid Metabolism, Small Molecule Biochemistry, Molecular Transport	43

(c) 2000-2023 QIAGEN. All rights reserved.

INGENUITY PATHWAY ANALYSIS

**2**	Connective Tissue Disorders, Developmental Disorder, Organismal Injury and Abnormalities	41
**3**	Hereditary Disorder, Metabolic Disease, Organismal Injury and Abnormalities	41
**4**	Developmental Disorder, Hereditary Disorder, Neurological Disease	41
**5**	Carbohydrate Metabolism, Cell Morphology, Connective Tissue Development and Function	41

Top Tox Lists

Name	p-value	Overlap
**Renal Necrosis/Cell Death**	2,00E-10	16,1 % 107/663
**Liver Proliferation**	5,11E-08	18,9 % 53/281
**Liver Necrosis/Cell Death**	9,38E-08	17,5 % 61/349
**Cardiac Necrosis/Cell Death**	1,35E-06	16,7 % 56/335
**Decreases Transmembrane Potential of Mitochondria and Mitochondrial Membrane**	1,97E-06	21,4 % 31/145

Top My Lists

(c) 2000-2023 QIAGEN. All rights reserved.

INGENUITY PATHWAY ANALYSIS

Top My Pathways

Top Analysis-Ready Molecules

Expr Log Ratio

Molecules	Expr. Value	Chart
SHH	↑ 3,694	
SLC22A3	↑ 3,643	
KCNK3	↑ 3,634	
HTR1D	↑ 3,618	
TTLL6	↑ 3,392	
ADGRG1	↑ 3,237	
ALS2CL	↑ 3,172	
SMCO2	↑ 3,170	
TINAGL1	↑ 3,123	
TFPT	↑ 2,998	

Expr Log Ratio

Molecules	Expr. Value	Chart
CECR2	↓ -4,103	
RBM11	↓ -3,962	

(c) 2000-2023 QIAGEN. All rights reserved.

INGENUITY PATHWAY ANALYSIS

**TNFSF18**	↓ -3,808
**HBA1/HBA2***	↓ -3,792
**DMRTA1**	↓ -3,240
**ILDR2**	↓ -3,074
**KLHDC7B**	↓ -2,978
**RASSF9**	↓ -2,941
**KLHL4**	↓ -2,874
**CADM3**	↓ -2,839

(c) 2000-2023 QIAGEN. All rights reserved.

INGENUITY PATHWAY ANALYSIS

INGENUITY
PATHWAY ANALYSIS

Analysis Name: SA1A - Monoculture - NRKE (with homologs) - 2023-02-16 11:20 vorm.
Analysis Creation Date: 2023-02-16
Build version: exported
Content version: 84978992 (Release Date: 2022-11-27)

Experiment Metadata

Name	Value

Analysis Settings

Reference set: Ingenuity Knowledge Base (Genes Only)
Relationship to include: Direct and Indirect
Includes Endogenous Chemicals
Optional Analyses: My Pathways My List

Filter Summary:
Consider only molecules and/or relationships where
(species = Uncategorized OR Human OR Mouse OR Rat) AND
(confidence = Experimentally Observed) AND
(tissues/cell lines = Other Dendritic cells OR Corpus Callosum OR Pancreas OR Other Melanoma Cell Lines OR Other Cell Line OR Hematopoietic progenitor cells OR Murine NKT cells OR SN12C OR U251 OR Central memory helper T cells OR Stromal cells OR White Matter OR Neutrophils OR Placenta OR Other Lymphoma Cell Lines OR Cornea OR NCI-H23 OR Other Smooth muscle cells OR Cos-7 cells OR SF-

(c) 2000-2023 QIAGEN. All rights reserved.

268 OR CD34+ cells OR Th2 cells OR Vd2 Gamma-delta T cells OR Memory T lymphocytes not otherwise specified OR Chondrocytes OR Skeletal Muscle OR Striatum OR HT29 OR Central memory cytotoxic T cells OR Trigeminal Ganglion OR J-774A.1 OR A549-ATCC OR Nucleus Accumbens OR Plasma cells OR A375 OR Granule cells OR SK-MEL-2 OR HepG2 OR Forestomach OR Amygdala OR Cardiomyocytes OR Granulocytes not otherwise specified OR SF-295 OR CD56dim NK cells OR Spleen OR RBL-2H3 OR CAKI-1 OR MCF7 OR Activated helper T cells OR Granulosa cells OR Activated Vd1 Gamma-delta T cells OR T lymphocytes not otherwise specified OR Colon Cancer Cell Lines not otherwise specified OR Other Breast Cancer Cell Lines OR Brainstem OR Cells not otherwise specified OR Peripheral blood monocytes OR Other Monocyte-derived dendritic cells OR NB4 OR Medulla Oblongata OR WEHI-231 OR Cytotoxic T cells OR BT-549 OR Lymphoma Cell Lines not otherwise specified OR Beta islet cells OR NCI-H332M OR Other Colon Cancer Cell Lines OR Other Neuroblastoma Cell Lines OR Other Stem cells OR Caudate Nucleus OR Melanocytes OR Other Cells OR Other Ovarian Cancer Cell Lines OR Monocyte-derived dendritic cells not otherwise specified OR Other Organ Systems OR Peripheral blood lymphocytes OR Other Hepatoma Cell Lines OR 3T3-L1 cells OR Other Bone marrow cells OR Fibroblasts OR HOP-92 OR Salivary Gland OR Large Intestine OR CNS Cell Lines not otherwise specified OR Bone marrow-derived macrophages OR Effector memory cytotoxic T cells OR Naive helper T cells OR UACC-62 OR OVCAR-8 OR OVCAR-4 OR MEF cells OR SK-MEL-5 OR Microvascular endothelial cells OR Other Prostate Cancer Cell Lines OR 786-0 OR Granule Cell Layer OR Lymphocytes not otherwise specified OR NCI-ADR-RES OR Dendritic cells not otherwise specified OR Stomach OR CD56bright NK cells OR Other Kidney cell lines OR Min6 OR Purkinje cells OR Activated CD56bright NK cells OR Brain OR Langerhans cells OR Mesenchymal stem cells OR Thalamus OR J774 OR Other CNS Cell Lines OR MDA-MB-468 OR UACC-257 OR Bone marrow cells not otherwise specified OR Other Nervous System OR MDA-MB-435 OR Jurkat OR RKO OR Macrophage Cancer Cell Lines not otherwise specified OR Cell Line not otherwise specified OR U266 OR Other Memory T lymphocytes OR Skin OR Monocyte-derived macrophage OR ACHN OR Other Granulocytes OR Melanoma Cell Lines not otherwise specified OR HCT-15 OR Pheochromocytoma cell lines not otherwise specified OR MOLT-4 OR RAW 264.7 OR Lung Cancer Cell Lines not otherwise specified OR SK-N-SH OR Other Pancreatic Cancer Cell Lines OR COLO205 OR Epithelial cells not otherwise specified OR Pro-B lymphocytes OR SK-OV-3 OR Testis OR Other Neurons OR RXF-393 OR Gray Matter OR Other NK cells OR BT-474 OR Olfactory Bulb OR Prostate Gland OR P19 OR Vd1 Gamma-delta T cells OR A2780 OR HS 578T OR Cortical neurons OR Other Osteosarcoma Cell Lines OR Megakaryocytes OR Memory B cells OR Kidney OR Trachea OR U937 OR Breast Cancer Cell Lines not otherwise specified OR LOX IMVI OR RPMI-8266 OR Natural T-regulatory cells OR MG-63 OR Th17 cells OR Oocytes OR Esophagus OR Other Endothelial cells OR TK-10 OR Monocytes not otherwise specified OR 293 cells OR SNB-75 OR Caco2 cells OR EKVX OR Other Lymphocytes OR Peritoneal macrophages OR Calvaria OR Other Immune cell lines OR Other Macrophage Cancer Cell Lines OR Hep3B OR Cervical cancer cell line not otherwise specified OR Prostate Cancer Cell Lines not otherwise specified OR T47-D OR Mammary Gland OR CD4+ T-lymphocytes OR Cerebellum OR Leukemia Cell Lines not otherwise specified OR CCRF-CEM OR KM-12 OR BDCA-1+ dendritic cells OR Hepatoma Cell Lines not otherwise specified OR Other Peripheral blood leukocytes OR U87MG OR Sciatic Nerve OR Other Macrophages

(c) 2000-2023 QIAGEN. All rights reserved.

OR Immune cell lines not otherwise specified OR LNCaP cells OR MDA-MB-231 OR HeLa OR HMC-1 OR UO-31 OR Crypt OR Activated Vd2 Gamma-delta T cells OR Naive B cells OR Parietal Lobe OR Dermis OR Thyroid Gland OR Other Pheochromocytoma cell lines OR NIH/3T3 cells OR Other T lymphocytes OR Cartilage Tissue OR Other Kidney Cancer Cell Lines OR PBMCs OR Adrenal Gland OR Effector memory RA+ cytotoxic T cells OR Heart OR Smooth Muscle OR Liver OR Osteoblasts OR Bladder OR Epidermis OR Mast cells OR Small Intestine OR Osteosarcoma Cell Lines not otherwise specified OR A498 OR Other Monocytes OR Peripheral blood leukocytes not otherwise specified OR Pituitary Gland OR Blood platelets OR THP-1 OR Macrophages not otherwise specified OR Myeloid dendritic cells OR Dorsal Root Ganglion OR HCC-2998 OR Adipose OR Cerebral Ventricles OR Pyramidal neurons OR NCI-H226 OR U2OS OR Myeloma Cell Lines not otherwise specified OR Substantia Nigra OR Cerebral Cortex OR Other Myeloma Cell Lines OR M14 OR Thymus OR DU-145 OR HL-60 OR Astrocytes OR B lymphocytes not otherwise specified OR Uterus OR PC-12 cells OR Vascular smooth muscle cells OR Th1 cells OR HuH7 OR Microglia OR Choroid Plexus OR Other Immune cells OR Spinal Cord OR Mature monocyte-derived dendritic cells OR Fibroblast cell lines not otherwise specified OR HCT-116 OR NT2/D1 OR Intraepithelial T lymphocytes OR Lung OR NCI-H522 OR PANC-1 OR Tissues and Primary Cells not otherwise specified OR Other Mononuclear leukocytes OR SW-480 OR BDCA-3+ dendritic cells OR Smooth muscle cells not otherwise specified OR SK-MEL-28 OR BA/F3 OR Ovarian Cancer Cell Lines not otherwise specified OR Other Epithelial cells OR Putamen OR Neurons not otherwise specified OR Effector T cells OR Other Tissues and Primary Cells OR Neuroblastoma Cell Lines not otherwise specified OR Pancreatic Cancer Cell Lines not otherwise specified OR MDA-MB-361 OR SR OR Bone marrow-derived dendritic cells OR Lens OR MALME-3M OR HOP-62 OR HEL OR Activated CD56dim NK cells OR Nervous System not otherwise specified OR Plasmacytoid dendritic cells OR Swiss 3T3 cells OR Endothelial cells not otherwise specified OR Hepatocytes OR Ventricular Zone OR PC-3 OR Immature monocyte-derived dendritic cells OR Immune cells not otherwise specified OR Retina OR Mononuclear leukocytes not otherwise specified OR Organ Systems not otherwise specified OR Other Lung Cancer Cell Lines OR Subventricular Zone OR H460 OR K-562 OR INS-1 OR Kidney cell lines not otherwise specified OR Hippocampus OR Teratocarcinoma Cell Lines not otherwise specified OR Sertoli cells OR Kidney Cancer Cell Lines not otherwise specified OR Effector memory helper T cells OR SF-539 OR Adipocytes OR MDA-N OR Eosinophils OR NK cells not otherwise specified OR Splenocytes OR OVCAR-3 OR SW-620 OR Ovary OR Embryonic stem cells OR HUVEC cells OR OVCAR-5 OR Hypothalamus OR Stem cells not otherwise specified OR Other Leukemia Cell Lines OR Other Teratocarcinoma Cell Lines OR Thymocytes OR Other Cervical cancer cell line OR Keratinocytes OR Pre-B lymphocytes OR Other B lymphocytes OR Other Fibroblast cell lines OR Lymph node OR IGROV1) AND
(mol. types = biologic drug OR canonical pathway OR chemical - endogenous mammalian OR chemical - endogenous non-mammalian OR chemical - kinase inhibitor OR chemical - other OR chemical - protease inhibitor OR chemical drug OR chemical reagent OR chemical toxicant OR complex OR cytokine OR disease OR enzyme OR function OR fusion gene/product OR G-protein coupled receptor OR group OR growth factor OR ion channel OR kinase OR ligand-dependent nuclear receptor OR mature microRNA OR microRNA OR other OR peptidase OR phosphatase OR transcription regulator OR translation regulator OR transmembrane receptor OR transporter) AND

(c) 2000-2023 QIAGEN. All rights reserved.

(data sources = An Open Access Database of Genome-wide Association Results OR BIND OR BioGRID OR Catalogue Of Somatic Mutations In Cancer (COSMIC) OR Chemical Carcinogenesis Research Information System (CCRIS) OR Clinical Genome Resource (ClinGen) OR ClinicalTrials.gov OR ClinVar OR Cognia OR DIP OR DrugBank OR Gene Ontology (GO) OR GVK Biosciences OR Hazardous Substances Data Bank (HSDB) OR HumanCyc OR Ingenuity Expert Findings OR Ingenuity ExpertAssist Findings OR IntAct OR Interactome studies OR MIPS OR miRBase OR miRecords OR Mouse Genome Database (MGD) OR Obesity Gene Map Database OR Online Mendelian Inheritance in Man (OMIM) OR TarBase OR TargetScan Human OR TargetScan Mouse)

Top Canonical Pathways

Name	p-value	Overlap
**Molecular Mechanisms of Cancer**	1,32E-14	47,1 % 212/450
**CLEAR Signaling Pathway**	2,03E-14	51,6 % 147/285
**Myelination Signaling Pathway**	2,48E-12	48,3 % 158/327
**HER-2 Signaling in Breast Cancer**	2,38E-11	51,1 % 116/227
**Pulmonary Fibrosis Idiopathic Signaling Pathway**	1,83E-10	46,6 % 152/326

Top Upstream Regulators

Upstream Regulators

Name	p-value	Predicted Activation
**HNF4A**	3,19E-66	
**TP53**	4,20E-64	
**beta-estradiol**	1,54E-45	Inhibited

(c) 2000-2023 QIAGEN. All rights reserved.

INGENUITY PATHWAY ANALYSIS

ESR1	1,84E-35	
TGFB1	1,00E-34	Inhibited

Causal Network

Name	p-value	Predicted Activation
HEXIM1	3,04E-83	
FGF3	2,96E-72	
colcemid	4,41E-72	
Ep300/Pcaf	4,97E-72	
Jmy-p300	2,59E-71	

Top Diseases and Bio Functions

Diseases and Disorders

Name	p-value range	# Molecules
Cancer	1,62E-14 - 0,00E00	6860
Endocrine System Disorders	1,25E-14 - 0,00E00	5956
Organismal Injury and Abnormalities	1,62E-14 - 0,00E00	6946
Gastrointestinal Disease	1,30E-14 - 5,45E-262	6150
Neurological Disease	9,96E-15 - 1,65E-163	4916

Molecular and Cellular Functions

(c) 2000-2023 QIAGEN. All rights reserved.

INGENUITY PATHWAY ANALYSIS

Name	p-value range	# Molecules
**Cellular Assembly and Organization**	8,86E-15 - 1,31E-72	1602
**Cellular Function and Maintenance**	8,86E-15 - 1,31E-72	2304
**Cell Death and Survival**	8,05E-15 - 1,26E-60	2518
**Gene Expression**	4,12E-21 - 8,00E-52	1556
**Cell Morphology**	8,86E-15 - 1,64E-48	1302

Physiological System Development and Function

Name	p-value range	# Molecules
**Organismal Survival**	5,18E-20 - 6,29E-81	1867
**Organismal Development**	1,59E-14 - 2,02E-34	2674
**Nervous System Development and Function**	8,86E-15 - 6,27E-34	1307
**Tissue Development**	1,59E-14 - 6,27E-34	2014
**Embryonic Development**	8,86E-15 - 4,61E-32	1726

Top Tox Functions

Assays: Clinical Chemistry and Hematology

Name	p-value range	# Molecules
**Increased Levels of Red Blood Cells**	3,49E-01 - 3,07E-06	59
**Increased Levels of Hematocrit**	3,50E-04 - 3,50E-04	47

(c) 2000-2023 QIAGEN. All rights reserved.

INGENUITY PATHWAY ANALYSIS

**Increased Levels of Alkaline Phosphatase**	4,73E-01 - 1,24E-03	40
**Decreased Levels of Albumin**	1,00E00 - 3,94E-02	17
**Decreased Levels of Hematocrit**	4,78E-02 - 4,78E-02	6

Cardiotoxicity

Name	p-value range	# Molecules
**Cardiac Enlargement**	1,00E00 - 5,25E-11	363
**Congenital Heart Anomaly**	1,00E00 - 7,38E-09	183
**Cardiac Fibrosis**	1,00E00 - 4,97E-08	150
**Cardiac Necrosis/Cell Death**	1,00E00 - 1,93E-07	142
**Cardiac Dysfunction**	1,00E00 - 2,96E-05	159

Hepatotoxicity

Name	p-value range	# Molecules
**Liver Hyperplasia/Hyperproliferation**	1,00E00 - 4,73E-61	3076
**Hepatocellular carcinoma**	1,00E00 - 1,30E-14	890
**Liver Necrosis/Cell Death**	1,00E00 - 1,62E-08	154
**Liver Steatosis**	1,00E00 - 7,18E-08	240
**Liver Proliferation**	5,11E-01 - 3,83E-07	127

Nephrotoxicity

(c) 2000-2023 QIAGEN. All rights reserved.

Name	p-value range	# Molecules
**Renal Necrosis/Cell Death**	1,00E00 - 3,33E-14	310
**Nephrosis**	1,00E00 - 1,31E-07	99
**Renal Proliferation**	5,81E-01 - 4,41E-06	139
**Renal Damage**	1,00E00 - 5,89E-05	152
**Renal Tubule Injury**	3,01E-01 - 5,89E-05	71

Top Regulator Effect Networks

ID	Regulators	Disease & Functions	Consistency Score
**1**	P2RY2	Cell movement	3,464
**2**	P2RY2	Migration of cells	3,464
**3**	let-7a-5p (and other miRNAs w/seed GAGGUAG)	Formation of filaments	3,328
**4**	THZ1	Size of body	3,317
**5**	actinomycin D	Formation of actin filaments	3,317

Top Networks

ID	Associated Network Functions	Score
**1**	Cell Signaling, Cell Morphology, Cellular Assembly and Organization	24
**2**	Hereditary Disorder, Metabolic Disease, Organismal Injury and Abnormalities	24

(c) 2000-2023 QIAGEN. All rights reserved.

INGENUITY PATHWAY ANALYSIS

3	DNA Replication, Recombination, and Repair, Developmental Disorder, Hereditary Disorder	24
4	RNA Post-Transcriptional Modification, Endocrine System Disorders, Gastrointestinal Disease	24
5	RNA Post-Transcriptional Modification, Cell-To-Cell Signaling and Interaction, Cellular Movement	24

Top Tox Lists

Name	p-value	Overlap	
**Renal Necrosis/Cell Death**	8,09E-20	46,6 %	309/663
**Cardiac Hypertrophy**	5,24E-09	43,9 %	169/385
**Cardiac Fibrosis**	5,53E-09	45,0 %	149/331
**Liver Necrosis/Cell Death**	1,62E-08	44,1 %	154/349
**Liver Proliferation**	5,41E-08	45,2 %	127/281

Top My Lists

(c) 2000-2023 QIAGEN. All rights reserved.

INGENUITY PATHWAY ANALYSIS

Top My Pathways

Top Analysis-Ready Molecules

Expr Log Ratio

Molecules	Expr. Value	Chart
COL2A1	↑ 7,071	
SPATC1L	↑ 6,997	
ALDH1L1	↑ 6,488	
SLC6A20	↑ 6,202	
ADAMTSL2	↑ 5,982	
PGLYRP2	↑ 5,933	
SIGLEC10	↑ 5,507	
FITM1	↑ 5,342	
DAND5	↑ 5,183	
ALDH1L2	↑ 5,120	

Expr Log Ratio

Molecules	Expr. Value	Chart
CXCL12	↓ -6,943	
PRRX1	↓ -6,638	
PDGFRA	↓ -6,248	
SFRP4	↓ -6,217	

(c) 2000-2023 QIAGEN. All rights reserved.

INGENUITY PATHWAY ANALYSIS

**VCAN**	↓ -6,152
**SLC17A6**	↓ -6,138
**OTC**	↓ -6,132
**Gm4756**	↓ -6,067
**LUM**	↓ -5,930
**GGT6**	↓ -5,789

(c) 2000-2023 QIAGEN. All rights reserved.

INGENUITY PATHWAY ANALYSIS

Analysis Name: SA1A - Monoculture - NRKF (with homologs) - 2023-02-16 11:26 vorm.
Analysis Creation Date: 2023-02-16
Build version: exported
Content version: 84978992 (Release Date: 2022-11-27)

Experiment Metadata

Name	Value

Analysis Settings

Reference set: Ingenuity Knowledge Base (Genes Only)
Relationship to include: Direct and Indirect
Includes Endogenous Chemicals
Optional Analyses: My Pathways My List

Filter Summary:
Consider only molecules and/or relationships where
(species = Uncategorized OR Human OR Mouse OR Rat) AND
(confidence = Experimentally Observed) AND
(tissues/cell lines = Other Dendritic cells OR Corpus Callosum OR Pancreas OR Other Melanoma Cell Lines OR Other Cell Line OR Hematopoietic progenitor cells OR Murine NKT cells OR SN12C OR U251 OR Central memory helper T cells OR Stromal cells OR White Matter OR Neutrophils OR Placenta OR Other Lymphoma Cell Lines OR Cornea OR NCI-H23 OR Other Smooth muscle cells OR Cos-7 cells OR SF-

(c) 2000-2023 QIAGEN. All rights reserved.

268 OR CD34+ cells OR Th2 cells OR Vd2 Gamma-delta T cells OR Memory T lymphocytes not otherwise specified OR Chondrocytes OR Skeletal Muscle OR Striatum OR HT29 OR Central memory cytotoxic T cells OR Trigeminal Ganglion OR J-774A.1 OR A549-ATCC OR Nucleus Accumbens OR Plasma cells OR A375 OR Granule cells OR SK-MEL-2 OR HepG2 OR Forestomach OR Amygdala OR Cardiomyocytes OR Granulocytes not otherwise specified OR SF-295 OR CD56dim NK cells OR Spleen OR RBL-2H3 OR CAKI-1 OR MCF7 OR Activated helper T cells OR Granulosa cells OR Activated Vd1 Gamma-delta T cells OR T lymphocytes not otherwise specified OR Colon Cancer Cell Lines not otherwise specified OR Other Breast Cancer Cell Lines OR Brainstem OR Cells not otherwise specified OR Peripheral blood monocytes OR Other Monocyte-derived dendritic cells OR NB4 OR Medulla Oblongata OR WEHI-231 OR Cytotoxic T cells OR BT-549 OR Lymphoma Cell Lines not otherwise specified OR Beta islet cells OR NCI-H332M OR Other Colon Cancer Cell Lines OR Other Neuroblastoma Cell Lines OR Other Stem cells OR Caudate Nucleus OR Melanocytes OR Other Cells OR Other Ovarian Cancer Cell Lines OR Monocyte-derived dendritic cells not otherwise specified OR Other Organ Systems OR Peripheral blood lymphocytes OR Other Hepatoma Cell Lines OR 3T3-L1 cells OR Other Bone marrow cells OR Fibroblasts OR HOP-92 OR Salivary Gland OR Large Intestine OR CNS Cell Lines not otherwise specified OR Bone marrow-derived macrophages OR Effector memory cytotoxic T cells OR Naive helper T cells OR UACC-62 OR OVCAR-8 OR OVCAR-4 OR MEF cells OR SK-MEL-5 OR Microvascular endothelial cells OR Other Prostate Cancer Cell Lines OR 786-0 OR Granule Cell Layer OR Lymphocytes not otherwise specified OR NCI-ADR-RES OR Dendritic cells not otherwise specified OR Stomach OR CD56bright NK cells OR Other Kidney cell lines OR Min6 OR Purkinje cells OR Activated CD56bright NK cells OR Brain OR Langerhans cells OR Mesenchymal stem cells OR Thalamus OR J774 OR Other CNS Cell Lines OR MDA-MB-468 OR UACC-257 OR Bone marrow cells not otherwise specified OR Other Nervous System OR MDA-MB-435 OR Jurkat OR RKO OR Macrophage Cancer Cell Lines not otherwise specified OR Cell Line not otherwise specified OR U266 OR Other Memory T lymphocytes OR Skin OR Monocyte-derived macrophage OR ACHN OR Other Granulocytes OR Melanoma Cell Lines not otherwise specified OR HCT-15 OR Pheochromocytoma cell lines not otherwise specified OR MOLT-4 OR RAW 264.7 OR Lung Cancer Cell Lines not otherwise specified OR SK-N-SH OR Other Pancreatic Cancer Cell Lines OR COLO205 OR Epithelial cells not otherwise specified OR Pro-B lymphocytes OR SK-OV-3 OR Testis OR Other Neurons OR RXF-393 OR Gray Matter OR Other NK cells OR BT-474 OR Olfactory Bulb OR Prostate Gland OR P19 OR Vd1 Gamma-delta T cells OR A2780 OR HS 578T OR Cortical neurons OR Other Osteosarcoma Cell Lines OR Megakaryocytes OR Memory B cells OR Kidney OR Trachea OR U937 OR Breast Cancer Cell Lines not otherwise specified OR LOX IMVI OR RPMI-8266 OR Natural T-regulatory cells OR MG-63 OR Th17 cells OR Oocytes OR Esophagus OR Other Endothelial cells OR TK-10 OR Monocytes not otherwise specified OR 293 cells OR SNB-75 OR Caco2 cells OR EKVX OR Other Lymphocytes OR Peritoneal macrophages OR Calvaria OR Other Immune cell lines OR Other Macrophage Cancer Cell Lines OR Hep3B OR Cervical cancer cell line not otherwise specified OR Prostate Cancer Cell Lines not otherwise specified OR T47-D OR Mammary Gland OR CD4+ T-lymphocytes OR Cerebellum OR Leukemia Cell Lines not otherwise specified OR CCRF-CEM OR KM-12 OR BDCA-1+ dendritic cells OR Hepatoma Cell Lines not otherwise specified OR Other Peripheral blood leukocytes OR U87MG OR Sciatic Nerve OR Other Macrophages

(c) 2000-2023 QIAGEN. All rights reserved.

INGENUITY PATHWAY ANALYSIS

OR Immune cell lines not otherwise specified OR LNCaP cells OR MDA-MB-231 OR HeLa OR HMC-1 OR UO-31 OR Crypt OR Activated Vd2 Gamma-delta T cells OR Naive B cells OR Parietal Lobe OR Dermis OR Thyroid Gland OR Other Pheochromocytoma cell lines OR NIH/3T3 cells OR Other T lymphocytes OR Cartilage Tissue OR Other Kidney Cancer Cell Lines OR PBMCs OR Adrenal Gland OR Effector memory RA+ cytotoxic T cells OR Heart OR Smooth Muscle OR Liver OR Osteoblasts OR Bladder OR Epidermis OR Mast cells OR Small Intestine OR Osteosarcoma Cell Lines not otherwise specified OR A498 OR Other Monocytes OR Peripheral blood leukocytes not otherwise specified OR Pituitary Gland OR Blood platelets OR THP-1 OR Macrophages not otherwise specified OR Myeloid dendritic cells OR Dorsal Root Ganglion OR HCC-2998 OR Adipose OR Cerebral Ventricles OR Pyramidal neurons OR NCI-H226 OR U2OS OR Myeloma Cell Lines not otherwise specified OR Substantia Nigra OR Cerebral Cortex OR Other Myeloma Cell Lines OR M14 OR Thymus OR DU-145 OR HL-60 OR Astrocytes OR B lymphocytes not otherwise specified OR Uterus OR PC-12 cells OR Vascular smooth muscle cells OR Th1 cells OR HuH7 OR Microglia OR Choroid Plexus OR Other Immune cells OR Spinal Cord OR Mature monocyte-derived dendritic cells OR Fibroblast cell lines not otherwise specified OR HCT-116 OR NT2/D1 OR Intraepithelial T lymphocytes OR Lung OR NCI-H522 OR PANC-1 OR Tissues and Primary Cells not otherwise specified OR Other Mononuclear leukocytes OR SW-480 OR BDCA-3+ dendritic cells OR Smooth muscle cells not otherwise specified OR SK-MEL-28 OR BA/F3 OR Ovarian Cancer Cell Lines not otherwise specified OR Other Epithelial cells OR Putamen OR Neurons not otherwise specified OR Effector T cells OR Other Tissues and Primary Cells OR Neuroblastoma Cell Lines not otherwise specified OR Pancreatic Cancer Cell Lines not otherwise specified OR MDA-MB-361 OR SR OR Bone marrow-derived dendritic cells OR Lens OR MALME-3M OR HOP-62 OR HEL OR Activated CD56dim NK cells OR Nervous System not otherwise specified OR Plasmacytoid dendritic cells OR Swiss 3T3 cells OR Endothelial cells not otherwise specified OR Hepatocytes OR Ventricular Zone OR PC-3 OR Immature monocyte-derived dendritic cells OR Immune cells not otherwise specified OR Retina OR Mononuclear leukocytes not otherwise specified OR Organ Systems not otherwise specified OR Other Lung Cancer Cell Lines OR Subventricular Zone OR H460 OR K-562 OR INS-1 OR Kidney cell lines not otherwise specified OR Hippocampus OR Teratocarcinoma Cell Lines not otherwise specified OR Sertoli cells OR Kidney Cancer Cell Lines not otherwise specified OR Effector memory helper T cells OR SF-539 OR Adipocytes OR MDA-N OR Eosinophils OR NK cells not otherwise specified OR Splenocytes OR OVCAR-3 OR SW-620 OR Ovary OR Embryonic stem cells OR HUVEC cells OR OVCAR-5 OR Hypothalamus OR Stem cells not otherwise specified OR Other Leukemia Cell Lines OR Other Teratocarcinoma Cell Lines OR Thymocytes OR Other Cervical cancer cell line OR Keratinocytes OR Pre-B lymphocytes OR Other B lymphocytes OR Other Fibroblast cell lines OR Lymph node OR IGROV1) AND
(mol. types = biologic drug OR canonical pathway OR chemical - endogenous mammalian OR chemical - endogenous non-mammalian OR chemical - kinase inhibitor OR chemical - other OR chemical - protease inhibitor OR chemical drug OR chemical reagent OR chemical toxicant OR complex OR cytokine OR disease OR enzyme OR function OR fusion gene/product OR G-protein coupled receptor OR group OR growth factor OR ion channel OR kinase OR ligand-dependent nuclear receptor OR mature microRNA OR microRNA OR other OR peptidase OR phosphatase OR transcription regulator OR translation regulator OR transmembrane receptor OR transporter) AND

(c) 2000-2023 QIAGEN. All rights reserved.

(data sources = An Open Access Database of Genome-wide Association Results OR BIND OR BioGRID OR Catalogue Of Somatic Mutations In Cancer (COSMIC) OR Chemical Carcinogenesis Research Information System (CCRIS) OR Clinical Genome Resource (ClinGen) OR ClinicalTrials.gov OR ClinVar OR Cognia OR DIP OR DrugBank OR Gene Ontology (GO) OR GVK Biosciences OR Hazardous Substances Data Bank (HSDB) OR HumanCyc OR Ingenuity Expert Findings OR Ingenuity ExpertAssist Findings OR IntAct OR Interactome studies OR MIPS OR miRBase OR miRecords OR Mouse Genome Database (MGD) OR Obesity Gene Map Database OR Online Mendelian Inheritance in Man (OMIM) OR TarBase OR TargetScan Human OR TargetScan Mouse)

Top Canonical Pathways

Name	p-value	Overlap
**Kinetochore Metaphase Signaling Pathway**	7,32E-11	41,4 % 46/111
**Axonal Guidance Signaling**	3,92E-10	26,3 % 134/509
**Senescence Pathway**	3,08E-09	29,1 % 87/299
**Myelination Signaling Pathway**	1,50E-08	27,8 % 91/327
**Pulmonary Fibrosis Idiopathic Signaling Pathway**	2,71E-08	27,6 % 90/326

Top Upstream Regulators

Upstream Regulators

Name	p-value	Predicted Activation
**TP53**	1,56E-53	Inhibited
**KRAS**	1,31E-37	Activated
**beta-estradiol**	1,79E-36	

(c) 2000-2023 QIAGEN. All rights reserved.

**TGFB1**	2,89E-32	Inhibited
**ESR1**	7,05E-31	

Causal Network

Name	p-value	Predicted Activation
**HEXIM1**	8,82E-68	Inhibited
**NCL**	1,62E-61	Inhibited
**RGS6**	4,77E-61	Inhibited
**SOX2**	8,57E-60	Inhibited
**CGM097**	1,15E-59	Activated

Top Diseases and Bio Functions

Diseases and Disorders

Name	p-value range	# Molecules
**Cancer**	4,53E-11 - 1,22E-207	3586
**Organismal Injury and Abnormalities**	4,91E-11 - 1,22E-207	3631
**Endocrine System Disorders**	1,76E-12 - 1,59E-162	3155
**Gastrointestinal Disease**	1,91E-11 - 3,56E-131	3241
**Neurological Disease**	3,56E-11 - 3,41E-81	2627

Molecular and Cellular Functions

(c) 2000-2023 QIAGEN. All rights reserved.

Name	p-value range	# Molecules
Cellular Assembly and Organization	3,57E-11 - 2,40E-44	1041
Cellular Function and Maintenance	3,97E-11 - 2,40E-44	1270
Cell Death and Survival	6,61E-12 - 6,54E-39	1342
Cell Cycle	4,17E-12 - 1,03E-30	677
Cellular Development	4,97E-11 - 1,32E-30	1416

Physiological System Development and Function

Name	p-value range	# Molecules
Organismal Survival	4,35E-12 - 1,01E-41	1002
Organismal Development	4,85E-11 - 2,39E-28	1494
Connective Tissue Development and Function	2,08E-12 - 4,75E-26	685
Tissue Development	1,98E-11 - 4,75E-26	1184
Cardiovascular System Development and Function	1,27E-11 - 2,07E-25	657

Top Tox Functions

Assays: Clinical Chemistry and Hematology

Name	p-value range	# Molecules
Increased Levels of Alkaline Phosphatase	1,78E-01 - 4,27E-06	31
Increased Levels of Hematocrit	6,31E-03 - 6,31E-03	26

(c) 2000-2023 QIAGEN. All rights reserved.

**Increased Levels of LDH**	1,00E00 - 1,51E-02	10
**Increased Levels of ALT**	1,00E00 - 2,50E-02	10
**Increased Levels of Creatinine**	4,27E-01 - 2,76E-02	17

Cardiotoxicity

Name	p-value range	# Molecules
**Cardiac Enlargement**	5,75E-01 - 3,04E-08	212
**Cardiac Necrosis/Cell Death**	6,42E-01 - 2,85E-07	87
**Cardiac Fibrosis**	1,00E00 - 1,25E-06	88
**Congenital Heart Anomaly**	1,00E00 - 2,81E-06	103
**Cardiac Dysfunction**	1,00E00 - 5,35E-05	92

Hepatotoxicity

Name	p-value range	# Molecules
**Liver Hyperplasia/Hyperproliferation**	1,00E00 - 9,44E-34	1641
**Hepatocellular carcinoma**	1,00E00 - 1,35E-15	518
**Liver Proliferation**	1,00E00 - 6,51E-09	83
**Liver Steatosis**	1,00E00 - 4,33E-07	140
**Liver Damage**	4,95E-01 - 7,92E-06	73

Nephrotoxicity

(c) 2000-2023 QIAGEN. All rights reserved.

INGENUITY PATHWAY ANALYSIS

Name	p-value range	# Molecules
**Renal Necrosis/Cell Death**	1,00E00 - 3,69E-10	170
**Renal Proliferation**	6,42E-01 - 2,23E-07	90
**Glomerular Injury**	1,00E00 - 2,79E-07	148
**Renal Damage**	4,95E-01 - 9,94E-07	99
**Renal Fibrosis**	2,90E-01 - 3,09E-05	47

Top Regulator Effect Networks

ID	Regulators	Disease & Functions	Consistency Score
1	COLQ,KCNJ2 (+6 more)	Atrial septal defect (+3 more)	12,964
2	EWSR1-FLI1,LTBP4,mir-133 (+11 more)	Atrial septal defect,Hypoplasia,Perinatal death (+1 more)	11,125
3	EWSR1-FLI1,LTBP4,MRTFB,SLC22A5,TEAD3	Hypoplasia,Organization of actin cytoskeleton (+1 more)	10,589
4	BMP15,GDF9,TXNRD1	Migration of endothelial cells (+2 more)	8,222
5	BACH1,CLEC4G,EZH2,GADD45GIP1,GSR,HDAC3,IL10RA,ILK (+11 more)	Fibrosis of liver	7,846

Top Networks

ID	Associated Network Functions	Score
1	Molecular Transport, RNA Trafficking, Cell Morphology	34

(c) 2000-2023 QIAGEN. All rights reserved.

INGENUITY PATHWAY ANALYSIS

**2**	DNA Replication, Recombination, and Repair, Gene Expression, Cell-To-Cell Signaling and Interaction	34
**3**	Cell-To-Cell Signaling and Interaction, Cellular Function and Maintenance, Inflammatory Response	32
**4**	Cancer, Gastrointestinal Disease, Organismal Injury and Abnormalities	32
**5**	Cellular Assembly and Organization, Developmental Disorder, Energy Production	32

Top Tox Lists

Name	p-value	Overlap
**Renal Necrosis/Cell Death**	1,77E-11	25,6 % 170/663
**Increases Glomerular Injury**	9,23E-11	35,9 % 61/170
**Liver Proliferation**	7,47E-09	29,2 % 82/281
**Acute Renal Failure Panel (Rat)**	1,74E-07	43,5 % 27/62
**Cardiac Fibrosis**	4,81E-07	26,3 % 87/331

Top My Lists

(c) 2000-2023 QIAGEN. All rights reserved.

INGENUITY PATHWAY ANALYSIS

Top My Pathways

Top Analysis-Ready Molecules

Expr Log Ratio

Molecules	Expr. Value	Chart
Rnase2 (includes others)*	↑ 4,820	
AMPD3	↑ 4,674	
COX6A2	↑ 4,634	
SPATA48	↑ 4,551	
SSTR3	↑ 3,999	
DISP2	↑ 3,984	
OLFM1	↑ 3,825	
FGFBP1	↑ 3,804	
PRSS22	↑ 3,790	
GDF15	↑ 3,785	

Expr Log Ratio

Molecules	Expr. Value	Chart
SIGLEC10	↓ -5,271	
CCDC177	↓ -4,902	

(c) 2000-2023 QIAGEN. All rights reserved.

MCEMP1	↓	-4,818
NAP1L3	↓	-4,563
GPR27	↓	-4,536
CHRNB4	↓	-4,359
CALHM5	↓	-4,272
Ms4a18	↓	-4,238
VNN1	↓	-4,229
PRKCZ	↓	-4,121

(c) 2000-2023 QIAGEN. All rights reserved.

INGENUITY PATHWAY ANALYSIS
