# Supplementary material for: Hypothesis-generating analysis of the impact of non-damaging metabolic acidosis on the transcriptome of different cell types: Integrated stress response (ISR) modulation as general transcriptomic reaction to non-respiratory acidic stress?
Source: PLoS One. 2023 Aug 25;18(8):e0290373. doi: 10.1371/journal.pone.0290373 (PMC10456223; doi:10.1371/journal.pone.0290373)
Supplement: S1 Fig — (PDF) [file pone.0290373.s002.pdf]

Supplementary Figure 1A: Graphical Summary of IPA analysis from HK-2 cells

SA1A - Monoculture - HK2 (with homologs) - 2023-02-16 10:59 vorm. Summary Graph

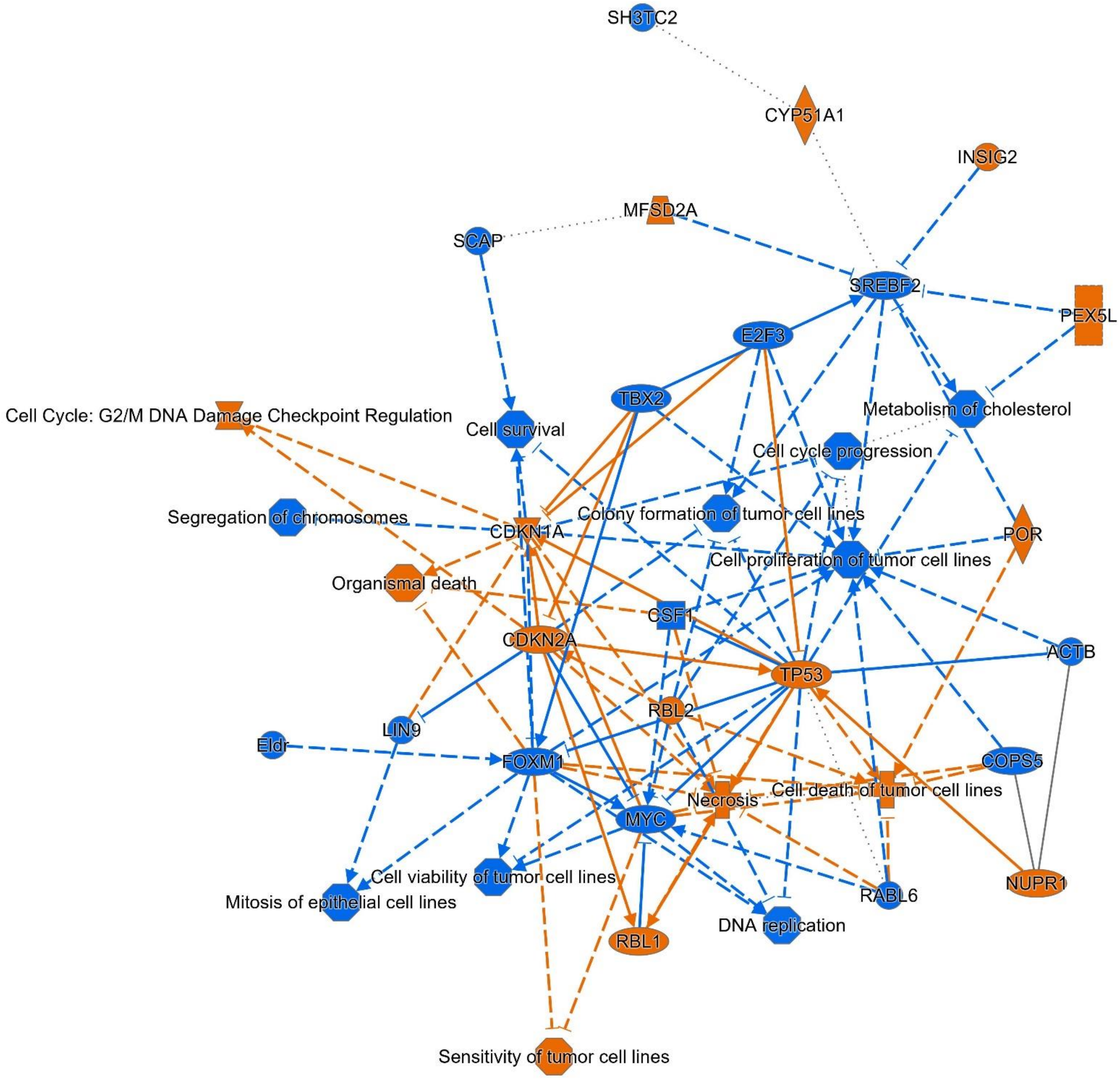

© 2000-2023 QIAGEN. All rights reserved.

Supplementary Figure 1B: Graphical Summary of IPA analysis from CCDKS cells

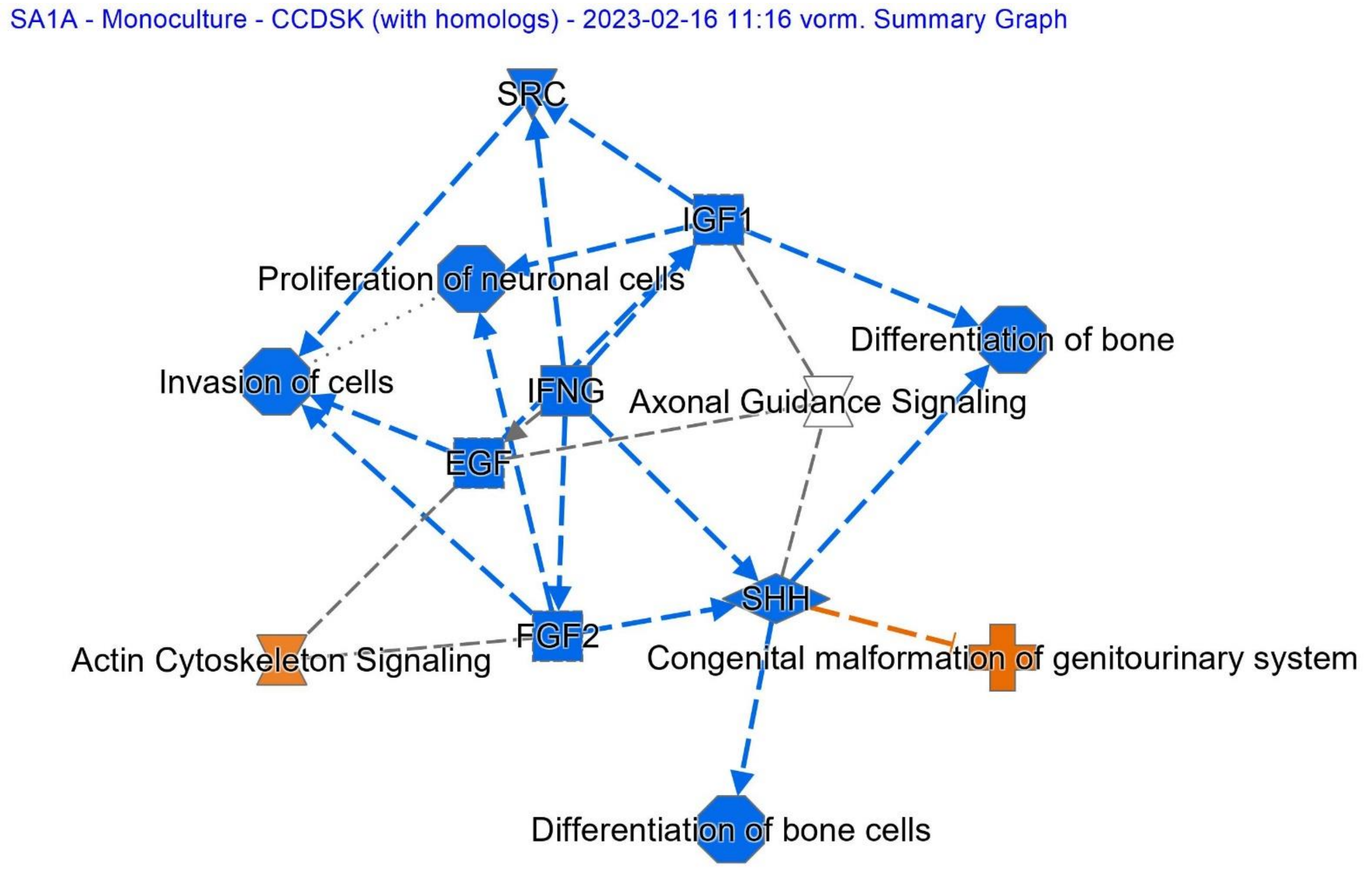

© 2000-2023 QIAGEN. All rights reserved.

Supplementary Figure 1C: Graphical Summary of IPA analysis from HAoSMC cells

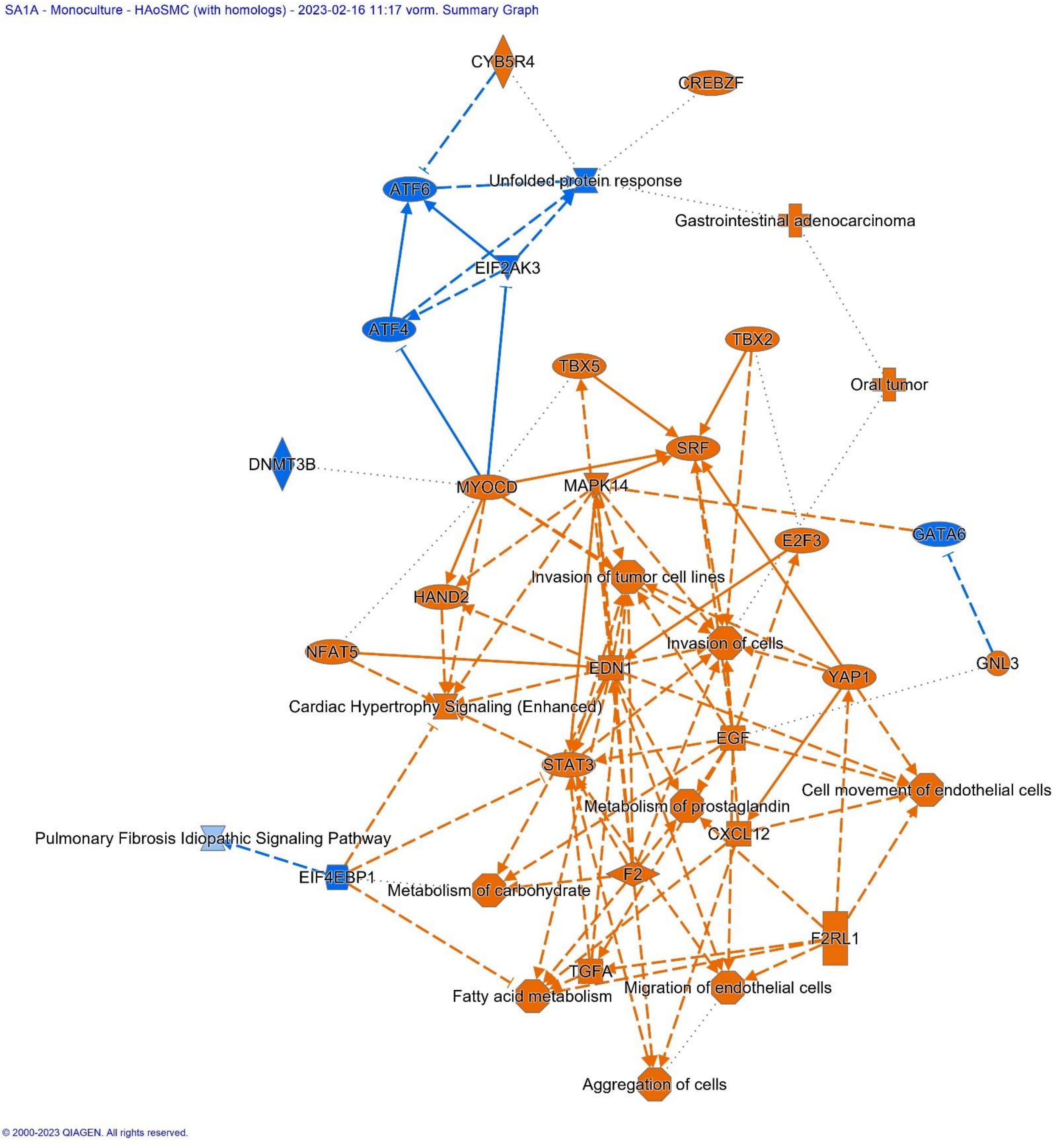

Supplementary Figure 1D: Graphical Summary of IPA analysis from NRK-52E cells

SA1A - Monoculture - NRKE (with homologs) - 2023-02-16 11:20 vorm. Summary Graph

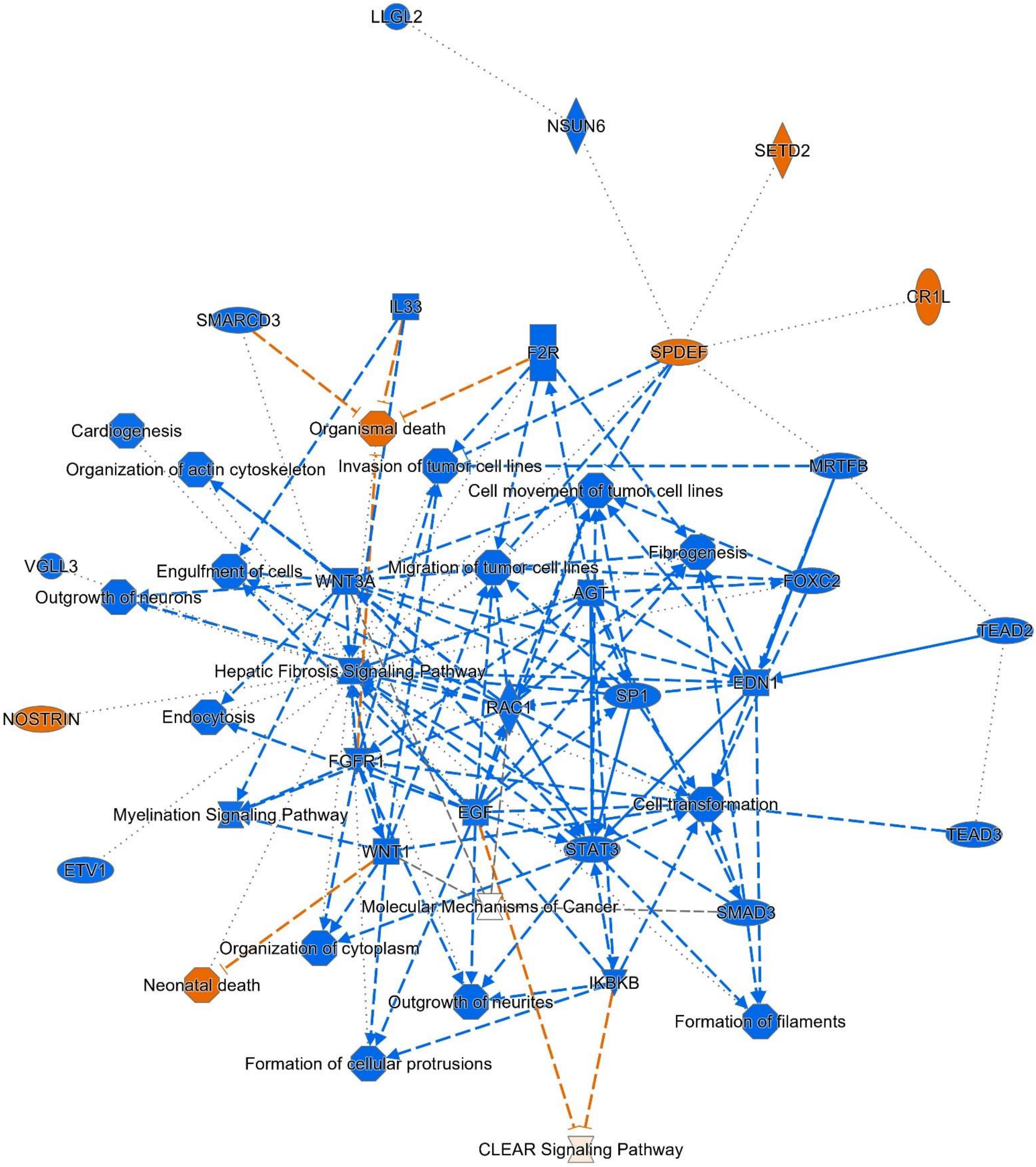

© 2000-2023 QIAGEN. All rights reserved.

Supplementary Figure 1E: Graphical Summary of IPA analysis from NRK-49F cells

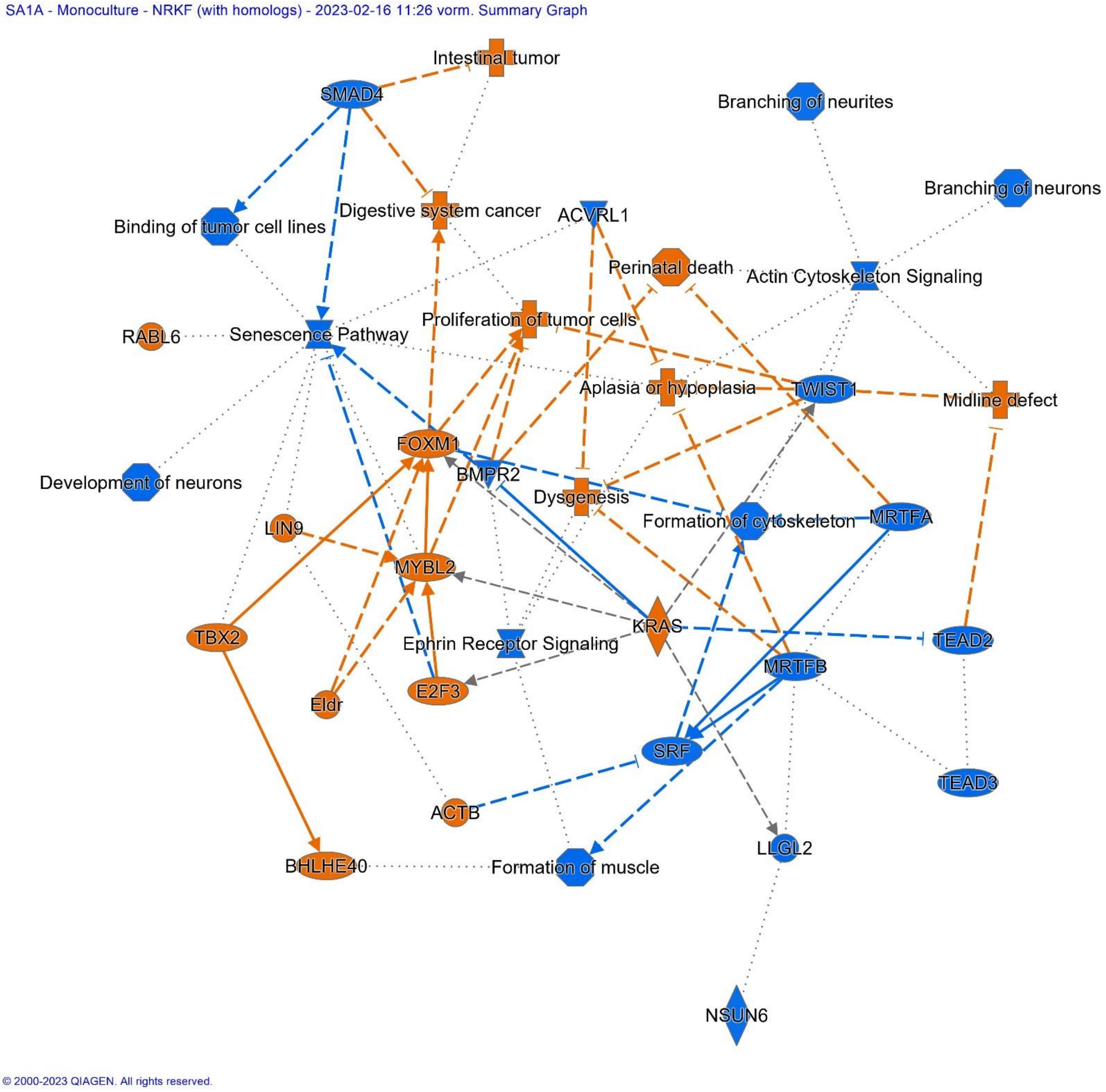

© 2000-2023 QIAGEN. All rights reserved.
