# Supplementary material for: Hypothesis-generating analysis of the impact of non-damaging metabolic acidosis on the transcriptome of different cell types: Integrated stress response (ISR) modulation as general transcriptomic reaction to non-respiratory acidic stress?
Source: PLoS One. 2023 Aug 25;18(8):e0290373. doi: 10.1371/journal.pone.0290373 (PMC10456223; doi:10.1371/journal.pone.0290373)

Supplementary Figure 2: Canonical pathway identification by IPA

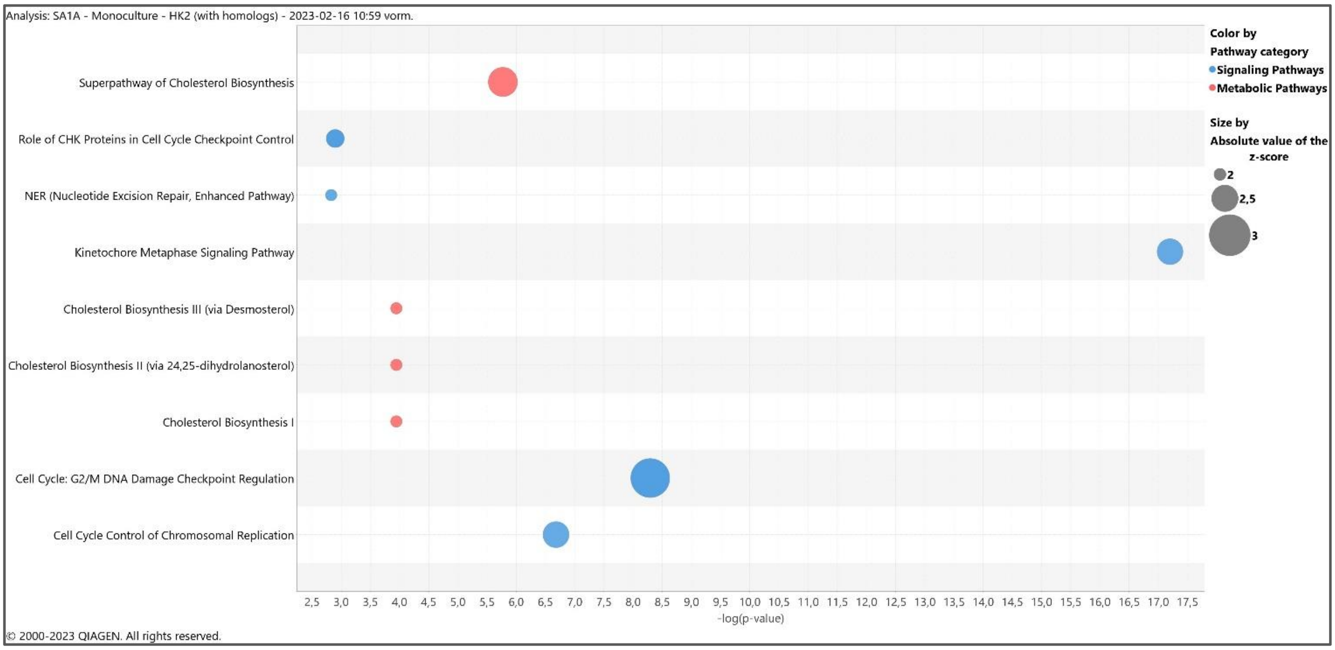

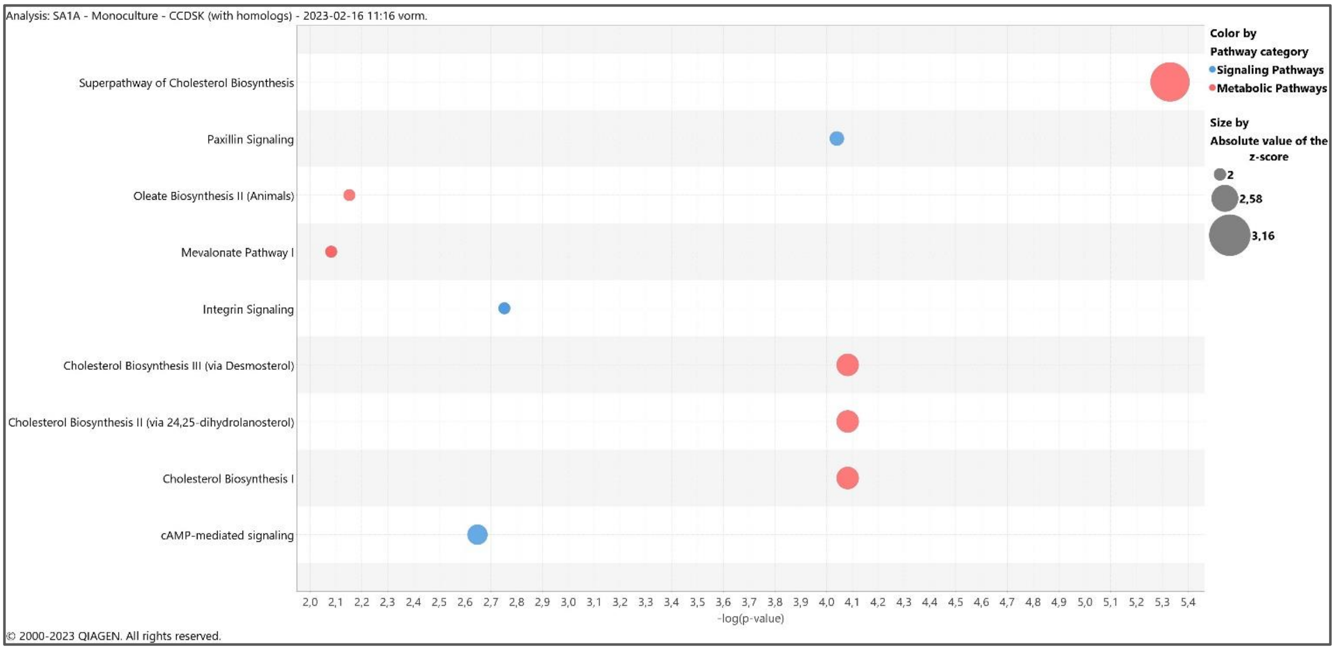

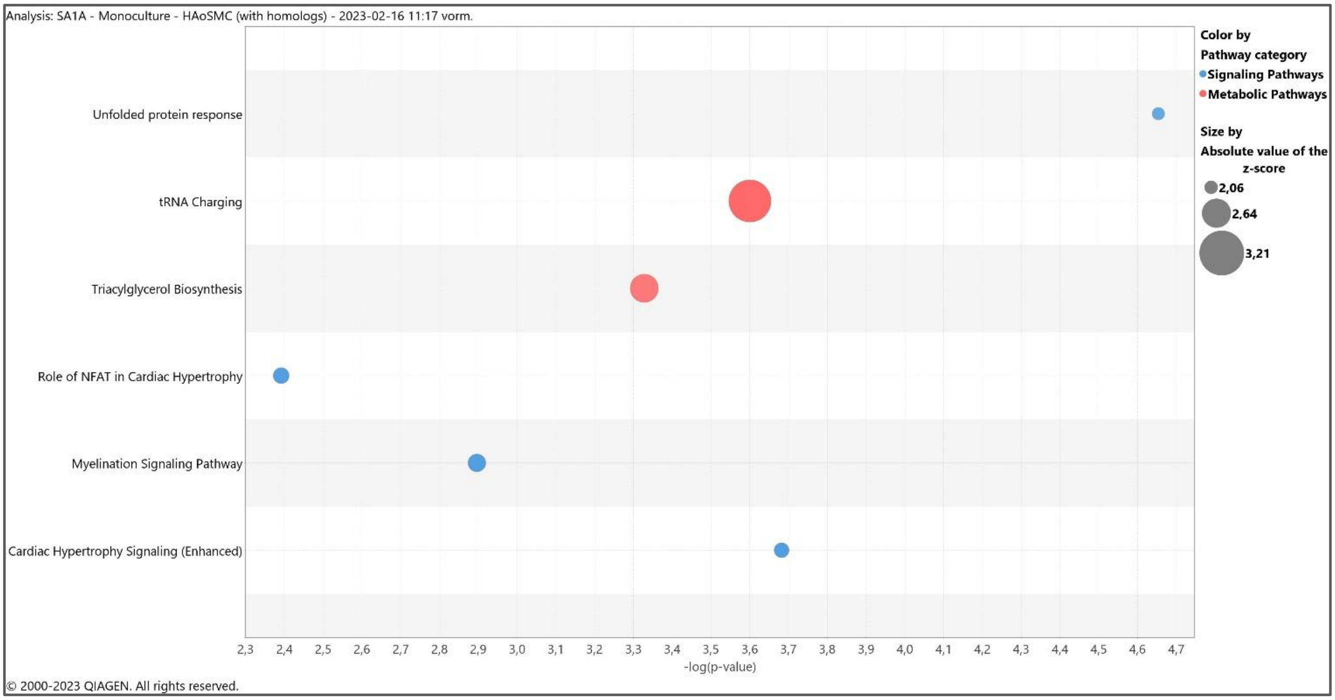

Supplementary Figure 2: Canonical pathway identification by IPA

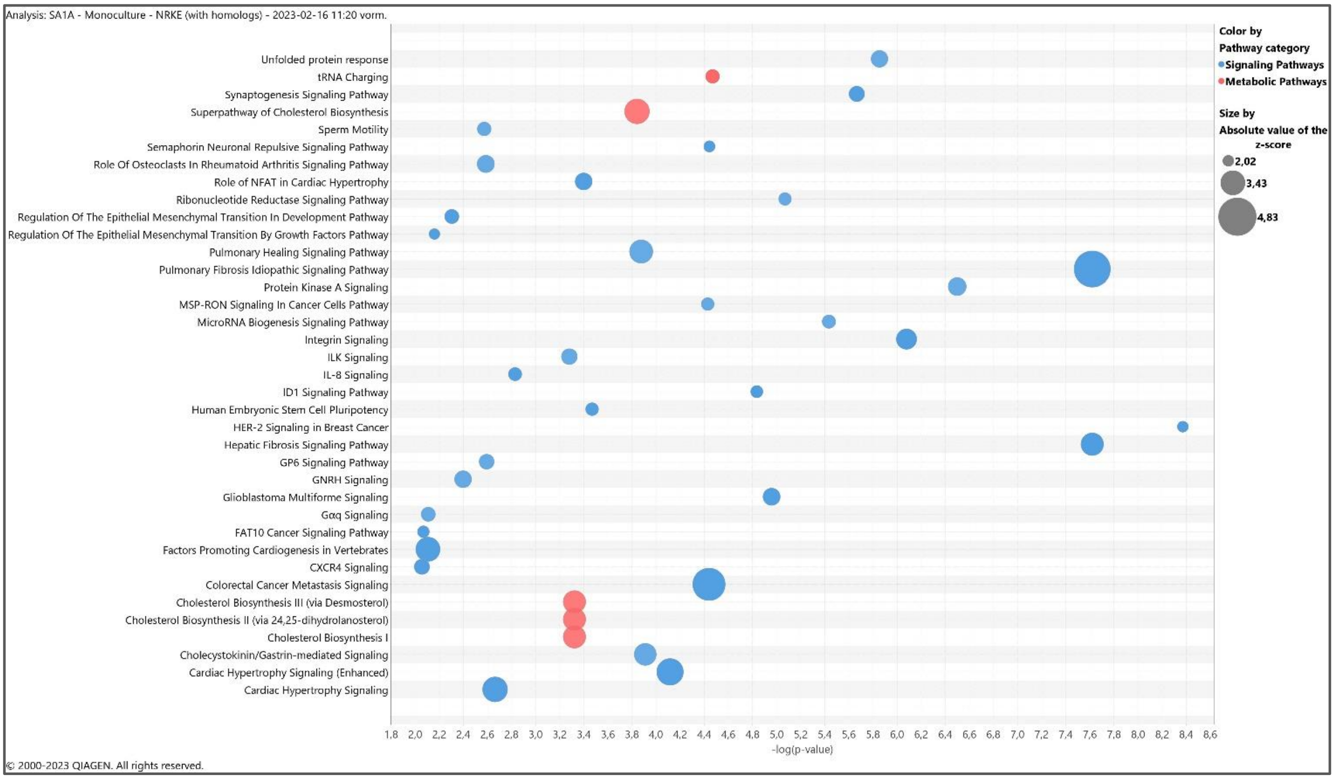

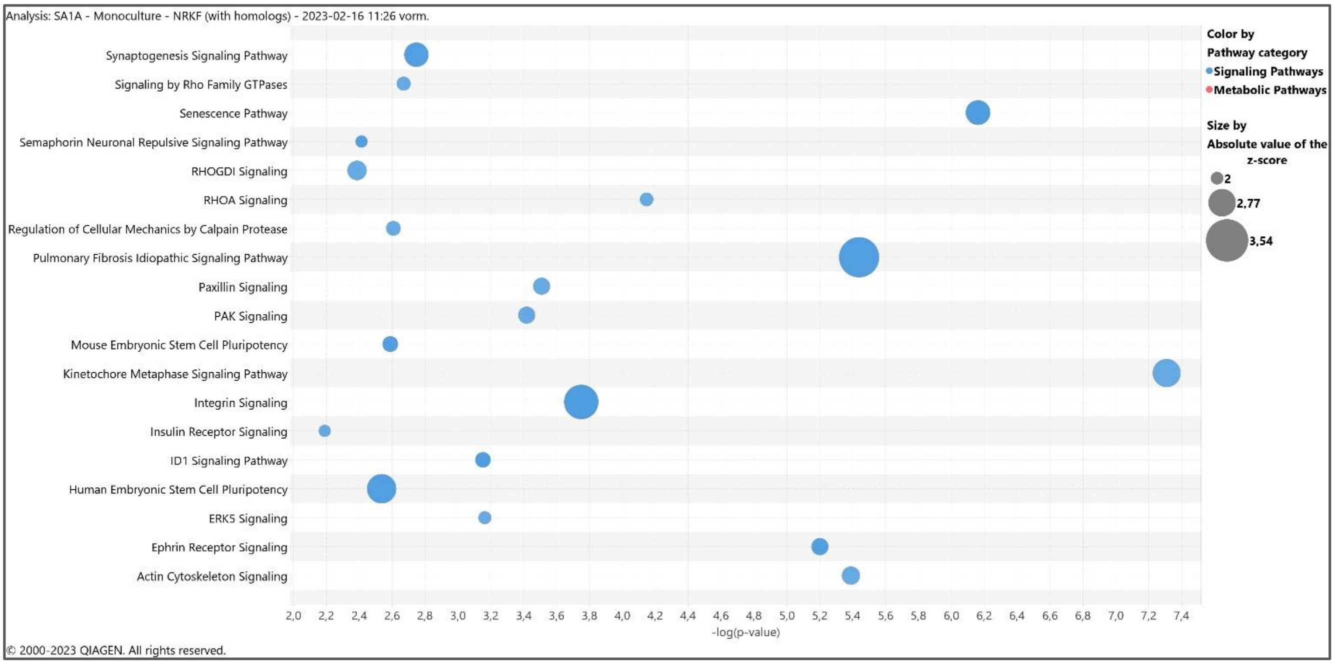

Supplement: S2 Fig — (PDF) [file pone.0290373.s003.pdf]
