# Supplementary material for: Hypothesis-generating analysis of the impact of non-damaging metabolic acidosis on the transcriptome of different cell types: Integrated stress response (ISR) modulation as general transcriptomic reaction to non-respiratory acidic stress?
Source: PLoS One. 2023 Aug 25;18(8):e0290373. doi: 10.1371/journal.pone.0290373 (PMC10456223; doi:10.1371/journal.pone.0290373)
Supplement: Attachment — Submitted filename: Response to Reviewers.pdf [file pone.0290373.s008.pdf]

MARTIN-LUTHER-UNIVERSITÄT
HALLE-WITTENBERG
Medizinische Fakultät

Julius-Bernstein-Institut für Physiologie
Direktor: Prof. Dr. med. Michael Gekle

Prof. Dr. M. Gekle, Julius-Bernstein-Institut für Physiologie,
Magdeburger Str. 6, 06112 Halle/Saale, Germany

To the Editor
PLoS ONE

Ihre Zeichen	Ihr Schreiben vom	Unsere Zeichen	Datum
			15.06.2023

Hausanschrift:
Magdeburger Str. 6
06112 Halle/Saale
Germany
Tel. (0345) 557 1886
Fax (0345) 557 4019

Sekretariat:
Kerstin Quarch

Tel.: (0345) 557 1886
Fax: (0345) 557 4019

e-mail:
kerstin.quarch@medizin.uni-halle.de

Internet:
www1.medizin.uni-halle.de/iph/

Direktor:
Prof. Dr. Michael Gekle

Tel.: (0345) 557 1886
Tel.: (0345) 557 1389
Fax: (0345) 557 4019

e-mail:
michael.gekle@medizin.uni-halle.de

Manuscript Submission

Dear Editor,

herewith we submit our revised manuscript entitled "**Hypothesis-generating analysis of the impact of non-damaging metabolic acidosis on the transcriptome of different cell types: Integrated stress response (ISR) modulation as general transcriptomic reaction to non-respiratory acidic stress?**"

We thank the reviewers for their comments and their recommendation to publish our manuscript. All comments raised by the reviewers and editorial office were addressed in the revised manuscript version, as detailed below.

Datasets generated and/or analyzed during the current study are available in the gene expression omnibus database. Human data: GSE220788 (token: glkxqmgclpofpiv). Rat data: GSE220789 (token: obcrcmkirbmbbgd).

All authors agree with the submission. The work has not been published or submitted for publication elsewhere. There is no conflict of interest.

Detailed responses **in bold** to the issues raised:

1. Please ensure that your manuscript meets PLOS ONE's style requirements, including those for file naming. The PLOS ONE style templates can be found at

https://journals.plos.org/plosone/s/file?id=wjVg/PLOSOne_formatting_sample_main_body.pdf and

https://journals.plos.org/plosone/s/file?id=ba62/PLOSOne_formatting_sample_title_authors_affiliations.pdf

We revised the manuscript according to the style templates above.

2. We suggest you thoroughly copyedit your manuscript for language usage, spelling, and grammar. If you do not know anyone who can help you do this, you may wish to consider employing a professional scientific editing service.

We went again thoroughly through the entire manuscript and checked it for grammar and spelling errors. As we are experienced in the use of the English language for scientific publications (and also spend time in Australia and the US), we think we are able to evaluate a scientific text concerning appropriate English writing.

A copy of our manuscript showing all changes by either using track changes is provided as a supporting information file.

A clean copy of the edited manuscript has been uploaded as the new manuscript file.

3. We noticed you have some minor occurrence of overlapping text with the following previous publication(s), which needs to be addressed:

We compared the text of the mentioned publications (3 of the mentioned publications are from our group) with the text of our manuscript, sentence by sentence, but could not detect major overlaps. Nevertheless, we modified few sentences in the introduction that showed some similarities due to the basic and general nature of the issues described.

https://journals.lww.com/jasn/pages/default.aspx

→ Although not specified we assume that it refers to following publication: Wesson DE, Buysse JM, Bushinsky DA (2020) Mechanisms of Metabolic Acidosis-Induced Kidney Injury in Chronic Kidney Disease. J Am Soc Nephrol 31: 469-482. The authors of this review describe, as we do in the introduction, mechanisms and cellular consequences of local acidosis. Because we were aware that similar issues are described for which the variability of wording is limited, we cited this review several times in the introduction (Ref. 3). We modified few sentences in the introduction that showed some similarities due to the basic and general nature of the issues described.

https://docksci.com/acidic-environment-activates-inflammatory-programs-in-fibroblasts-via-a-camp-map_5a7bbb2cd64ab22d4c439d9f.html

→ This is a publication of our group that also deals with the impact of acidosis. Therefore, a certain overlap in wording, especially in the introduction and the method section, may occur. However, we could not detect overlaps outside the method section. Ref 16. A. Riemann, … M. Gekle. Biochimica et Biophysica Acta 1853 (2015) 299–307.

https://pubs.acs.org/doi/10.1021/acs.jproteome.5b00503

→ This is a publication of our group that also deals with the impact of acidosis. Therefore, a certain overlap in wording, especially in the introduction especially in the introduction and the method section, may occur. However, we could not detect relevant overlaps outside the method section. Nevertheless, we modified one sentence. A. Ihling, … M. Gekle. J. Proteome Res. 2015, 14, 9, 3996–4004

https://www.mdpi.com/2076-3921/12/2/412

→ This is a publication of our group that also deals with the impact of acidosis. Therefore, a certain overlap in wording, especially in the introduction especially in the introduction and the method section, may occur. However, we could not detect overlaps outside the method section. Ref. 13. MC Schulz, … M. Gekle. Antioxidants 2023, 12(2), 412

https://academic.oup.com/hmg/article/26/R2/R91/3978054

→ This is a completely unrelated publication that is unknown to us. This study deals with a neuropathological topic, amyotrophic lateral sclerosis, which is not related to our area of expertise. In their manuscript the authors discuss the general cell biological mechanism of "integrated stress response" as we do and cite a publication that we also cite (Pakos-Zebrucka K, Koryga I, Mnich K, Ljujic M, Samali A, Gorman AM (2016) The integrated stress response. EMBO rep 17: 1374-1395.). Integrated stress response is a clearly defined cellular mechanism, the characterization of which does not allow room for diverse explanations. Therefore, you may find overlapping wording whenever integrated stress response is described or discussed. However, we could not identify overlapping wording in terms of partial or even entire phrases.

We checked our revision carefully concerning the appropriate citation of all sources.

4. We note that the grant information you provided in the 'Funding Information' and 'Financial Disclosure' sections do not match.

When you resubmit, please ensure that you provide the correct grant numbers for the awards you received for your study in the 'Funding Information' section.

We added the correct information: This project was supported funded by the Deutsche Forschungsgemeinschaft (DFG GE 905/19). The funders had no role in study design, data collection and analysis, decision to publish, or preparation of the manuscript.

5. We note that you have indicated that data from this study are available upon request.

This must be a misunderstanding, since we declared "Yes - all data are fully available without restriction". This is documented in the PONE-S-23-12894 file. Datasets generated and/or analyzed during the current study are available in the gene expression omnibus database. Human data: GSE220788 (token: glkxqmgclpofpiv). Rat data: GSE220789 (token: obcrcmkirbmbbgd).

6. Please include captions for your Supporting Information files at the end of your manuscript, and update any in-text citations to match accordingly. Please see our Supporting Information guidelines for more information: http://journals.plos.org/plosone/s/supporting-information.

Done.

7. Please review your reference list to ensure that it is complete and correct. If you have cited papers that have been retracted, please include the rationale for doing so in the manuscript text, or remove these references and replace them with relevant current references. Any changes to the reference list should be mentioned in the rebuttal letter that accompanies your revised manuscript. If you need to cite a retracted article, indicate the article's retracted status in the References list and also include a citation and full reference for the retraction notice.

Done.

Additional Editor Comments:

Please respond to the comment of the reviewer to validate the finding at RT-PCR level. **We performed a comprehensive bioinformatic analysis on RNA sequencing results comprising a large number of genes from five different cell types, to identify patterns and generate new hypothesis. This analysis includes bioinformatically derived upstream predictions concerning the activity of signaling modules as well as downstream predictions concerning cellular functions. These parameters cannot be validated by RT-PCR, but have to be investigated at the protein and/or functional level. These analyses are the focus of future projects and are beyond the scope of the present study. Furthermore, high quality RNA sequencing with the appropriate number of biological replicates followed by state–of-the-art differential expression analysis, including extensive quality control and normalization, leads to more reliable and valid results compared to RT-PCR with one or two housekeepers. We are convinced that the next step must be the determination of relevant alterations at the protein level (changes in expression or in modification, like phosphorylation) and/or functional cell assays. Currently we are setting up the methodology for these work packages.**

Reviewers' comments:

1. In page9, paragraph 3, line 7, research should be changed as researches.

The word "research" does not appear on page 9. On page 3 there is the phrase "In the past, research focused mainly on tumor microenvironment acidosis as key parameter for cell fate and disease progression." This phrase is grammatically correct.

2. In page11, paragraph 1, line 3, CO2 should be changed as CO2.

On page 5 CO2 was changed to CO_2.

3. In page22, Figure 4, line 1,shows should be changed as show.

Shows was changed to show.

4. I think RT-PCR experiments should be used to verify the RNS-seq data.

We performed a comprehensive bioinformatical analysis on RNA sequencing results comprising a large number of genes from five different cell types, to identify patterns and generate new hypothesis. This analysis includes bioinformatically derived upstream predictions concerning the activity of signaling modules as well as downstream predictions concerning cellular functions. These parameters cannot be validated by RT-PCR, but have to be investigated at the protein and/or functional level. These analyses are the focus of future project and are beyond the scope of the present study. Furthermore, high quality RNA sequencing with the appropriate number of biological replicates fol-

lowed by state-of-the art differential expression analysis, including extensive quality control and normalization, leads to more reliable and valid results compared to RT-PCR with one or two housekeepers. We are convinced that the next step must be the determination of relevant alterations at the protein level (changes in expression or in modification, like phosphorylation) and/or functional cell assays. Currently we are setting up the methodology for these work packages.

5. The English writing should be largely improved.

Both reviewers answered question 4 above ("Is the manuscript presented in an intelligible fashion and written in standard English?") with yes. Thus, the comment raised here is contradictory. As we are experienced in the use of the English language for scientific publications (and also spend time in Australia and the US), we think we are able to evaluate a scientific text concerning appropriate English writing. Nevertheless, we went again thoroughly through the entire manuscript and checked it for grammar and spelling errors.

Sincerely,

Prof. Dr. Michael Gekle
